# Enhancement of superconducting properties in the La–Ce–H system at moderate pressures

**DOI:** 10.1038/s41467-023-38254-6

**Published:** 2023-05-09

**Authors:** Wuhao Chen, Xiaoli Huang, Dmitrii V. Semenok, Su Chen, Di Zhou, Kexin Zhang, Artem R. Oganov, Tian Cui

**Affiliations:** 1 State Key Laboratory of Superhard Materials, College of Physics, Jilin University, Changchun, 130012 China; 2 Center for High Pressure Science and Technology Advanced Research (HPSTAR), Beijing, 100094, China; 3 Skolkovo Institute of Science and Technology, Skolkovo Innovation Center, Bolshoy Boulevard 30, bldg. 1, Moscow, 121205 Russia; 4 School of Physical Science and Technology, Ningbo University, Ningbo, 315211 China

**Keywords:** Superconducting properties and materials, Electronic properties and materials

## Abstract

Ternary hydrides are regarded as an important platform for exploring high-temperature superconductivity at relatively low pressures. Here, we successfully synthesized the *hcp*-(La,Ce)H_9-10_ at 113 GPa with the initial La/Ce ratio close to 3:1. The high-temperature superconductivity was strikingly observed at 176 K and 100 GPa with the extrapolated upper critical field *H*_c2_(0) reaching 235 T. We also studied the binary La-H system for comparison, which exhibited a *T*_c_ of 103 K at 78 GPa. The *T*_c_ and *H*_c2_(0) of the La-Ce-H are respectively enhanced by over 80 K and 100 T with respect to the binary La-H and Ce-H components. The experimental results and theoretical calculations indicate that the formation of the solid solution contributes not only to enhanced stability but also to superior superconducting properties. These results show how better superconductors can be engineered in the new hydrides by large addition of alloy-forming elements.

## Abstract (web summary)

Recently, high-temperature superconductivity has been reported in LaH_10_ and CeH_10_. Here, the authors report superconductivity in the alloy (La,Ce)H_9-10_ with *T*_c_ = 176 K at 100 GPa, providing an improved compromise between high transition temperature and low pressure requirements.

## Introduction

Recent discoveries of hydrogen-based superconductors, such as H_3_S^1–5^, LaH_10_^6–9^, YH_6_ and YH_9_^10–12^, CeH_9_ and CeH_10_^13–15^, and CaH_6_^16,17^ have inspired enormous interest in multiple areas. Binary high-temperature superconducting (HTSC) hydrides can be basically divided into two categories. The first theoretically and experimentally discovered hydride H_3_S belongs to the class of covalent polyhydrides^2,3^, which require covalent bonding between hydrogen and light nonmetal elements located in the upper right corner of the periodic table. The second type is mainly alkaline earth and rare earth metal polyhydrides with sodalite-like clathrate structures^16–18^, currently at the forefront of research. Besides, layered hydrides with graphene-like hydrogen nets are also predicted to have great potential as high-temperature superconductors^19^. Although the records of the superconducting *T*_c_ keep getting updates, obtaining HTSC phases at moderate pressures is still an elusive goal. More attention has been turned to ternary systems, typically for La-based HTSC polyhydrides^20–22^. The complexity of ternary hydrides in theory invites an experimental approach^23,24^. However, this is thwarted by poorly controlled and complex synthesis.

Superconducting binary hydrides of lanthanum^6–9,25–29^ and cerium^13–15,30–32^ have been well studied before and have the similar crystal structures. Lanthanum polyhydride *Fm*$\bar{3}$*m*-LaH_10_ holds the record of *T*_c_ (up to 250 K at 170 GPa), whereas cerium hydrides *P*6_3_/*mmc*-CeH_9_ and *Fm*$\bar{3}$*m*-CeH_10_ display HTSC properties at much lower pressures (*T*_c_ up to 115 K at 95 GPa). Alloying these two hydrides may lead to ternary hydrides with enhanced properties that can be stable at moderate pressures. As neighbors in the periodic table, La and Ce have very close atomic radii and electronegativities and can form continuous and homogeneous solid solutions^30,33,34^. In this work, we have synthesized ternary HTSC*-*(La,Ce)H_9-10_ with metal atoms forming the hexagonal close-packed (*P*6_3_/*mmc*) sublattice. Superconductivity is strikingly preserved to ∼100 GPa with *T*_c_ of 176 K and extrapolated *H*_c2_(0) of 235 T. This phase is stable under lower pressures with the giant enhancement of superconducting properties compared with the binary La–H and Ce–H system in the same pressure range. Present evidence suggests that the disordered state of (La,Ce)H_9-10_ has a significant effect on the stability and superconducting properties.

## Results

### Synthesis and characterization of the La–Ce alloys

We have chosen to synthesize ternary La–Ce–H hydride by a reaction of a La–Ce alloy with hydrogen, which is a relatively simple approach to synthesize multinary polyhydrides. Under pressure, the concentration of ~75% La is predicted to be the phase boundary in the La–Ce phase diagram that may be in favor of the lattice reconstruction^35^. Hence, we used the La_~0.75_Ce_~0.25_ alloy (the ratio of La–Ce is 3:1) prepared by multitarget magnetron sputtering as the initial reactant for typical experimental runs (Supplementary Table S1 and Fig. S1). Before loading into the diamond anvil cell, we have characterized this solid solution with different methods. We got the exact concentration by scanning electron microscopy equipped with energy-dispersive X-ray spectroscopy (SEM + EDX), which also proved the homogeneous distribution of the elements (Supplementary Figs. S14–S16 and S21). The X-ray diffraction (XRD) measurement revealed that the La–Ce alloy belonged to *Fm*$\bar{3}$*m* symmetry without any other impurity phase (Supplementary Fig. S2). We also discovered that the introduction of Ce atoms obviously suppressed the *T*_c_ of pure La. The single superconducting transition judging from the *R*–*T* curve partly indicated the homogeneity (Supplementary Figs. S19 and S20). It is noteworthy that La and Ce atoms retain random distribution over metal sites even after the formation of the polyhydride at high pressure.

### Superconductivity in the La–Ce–H and La–H systems

**Fig. 1:**
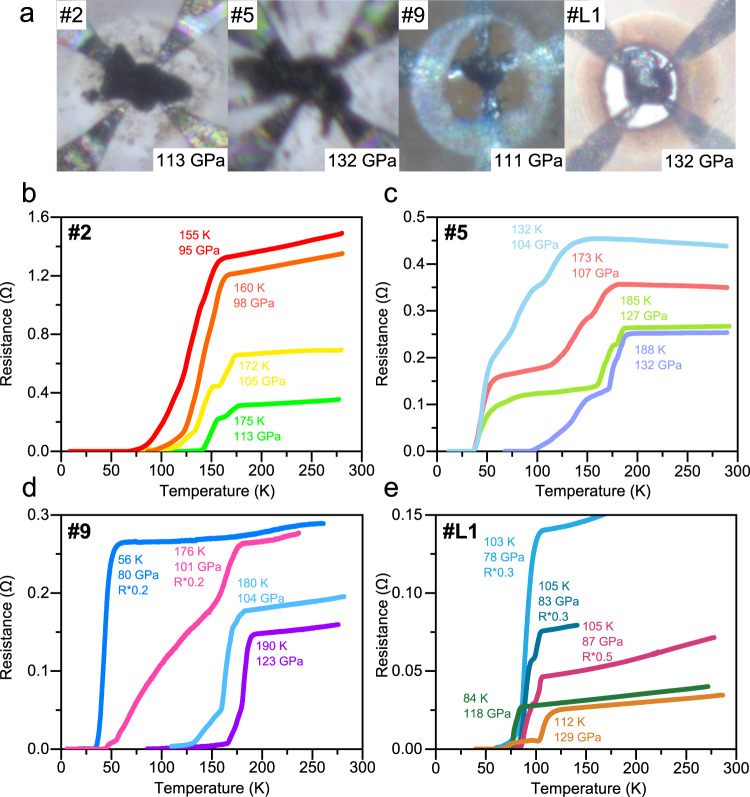
Characterization of the superconducting transitions using electrical resistance measurements at selected pressures for typical runs. **a** Photographs of the sample after laser heating together with four electrodes. **b**–**e** The temperature dependence of the electrical resistance for the La–Ce–H sample in DACs **#2,** **5,** **9** and La–H sample in DAC **#L1**.

To synthesize La–Ce–H or La–H compounds, we compressed the La–Ce alloy or pure La in the ammonia borane (NH_3_BH_3_) sample, which acted both as the pressure-transmitting medium and source of hydrogen. After that, the samples were laser-heated at the target pressure for a few seconds. In this process, hydrogen was released and reacted with La-Ce alloy or pure La at a temperature up to 1500 K, following which reaction products were quenched to room temperature. After synthesizing La–Ce–H compounds by laser heating at specific pressures, we conducted the electrical measurements and plotted the typical data in Fig. 1. To explore the possible high-temperature superconductivity at pressures lower than those of the known LaH_10_, we laser-heated the samples in DACs **#2** and **#9** at 113 GPa and 120 GPa, respectively. Strikingly, the *T*_c_ reaches 175 K (113 GPa, Fig. 1b) and 190 K (123 GPa, Fig. 1d), which is about 100 K higher than our previously discovered cerium polyhydrides^15^. The obtained products are expected to remain metastable below the synthetic pressure. To explore this, we measured the pressure dependence of *T*_c_ upon decompression and found *T*_c_ of 155 K at 95 GPa (DAC **#2**) and 180 K at 104 GPa (DAC **#9**). To compare with LaH_10_, we heated DAC **#6** at 152 GPa and observed the main resistance drop starting from 188 K (Supplementary Fig. S17). We also noticed another slight drop at 206 K. This is because of the formation of two superconducting phases (see Supplementary Fig. S18). *T*_c_ slightly decreases with further compression to 156 GPa and 162 GPa, which is different from the behavior of *C*2/*m*-LaH_10_^29^. Noteworthy, the pressure scale (diamond Raman edge) used in this study, as well as from Somayazulu et al.^8^, gives a higher value than using the hydrogen vibron by ~18 GPa^29^. The same diamond Raman edge scale has been used in both compression and decompression processes. DACs **#3** and **#5** were both laser-heated at about 130 GPa. Similar to sample **#6**, we also observed a slight resistance drop at 186 K for sample **#3** (Supplementary Fig. S8). Sample **#5** showed an obvious step-like transition which indicated the superconductivity of other phases at lower temperatures. The highest *T*_c_, decreasing gradually along with the pressure, dropped to 132 K at 104 GPa. In contrast, the lower *T*_c_ phase (~37 K, at the resistance close to zero) was robust during decompression from 127 GPa (Fig. 1c) to at least 80 GPa (Fig. 1d). Further decompression of sample **#9** from 123 to 101 GPa showed the tendency similar to that of the high-*T*_c_ phase in DACs **#2** and **#5** (Fig. 1b, c). The resistance of the La–Ce–H samples increased significantly when pressure decreased, and the width of the superconducting transition increased about 2.5 times from 104 GPa to 101 GPa (Fig. 1d), possibly due to disordering of the structure in the vicinity of a phase transition. Compared with the binary *fcc*-CeH_10_ and *hcp*-CeH_9_^15^, we obtained higher *T*_c_ in the ternary La–Ce–H system. However, there was no report on the superconductivity of binary La–H system at pressures lower than 120 GPa for our comparison. To fill this gap, we also explored the La–H system in the same pressure range (Fig. 1e). In DAC **#L1**, the La–H system showed the *T*_c_ of 84 K after the first laser heating at 123 GPa. Further increasing the pressure and laser heating the sample led to another superconducting transition with a higher *T*_c_ of 112 K at 129 GPa (Supplementary Fig. S30). During the decompression of DAC **#L1** (Supplementary Fig. S31), superconductivity was preserved down to 78 GPa with a *T*_c_ = 103 K (Fig. 1e and Supplementary Fig. S28). These data indicate that La–Ce–H system has a greatly enhanced *T*_c_ compared with La–H and Ce–H systems below 130 GPa.

### The structural analysis of the La–Ce–H and La–H systems

**Fig. 2:**
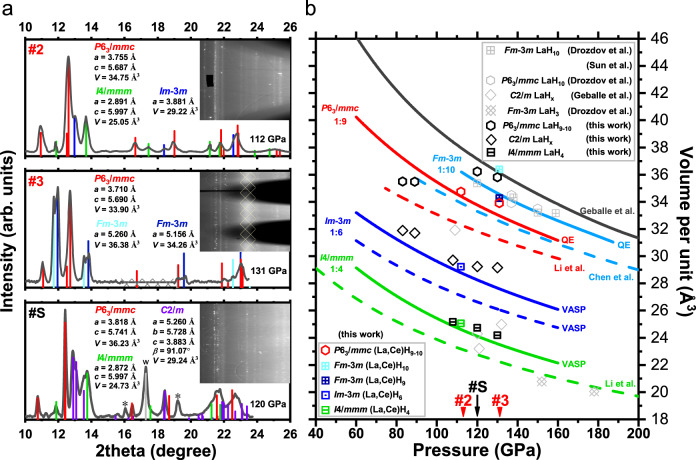
Synchrotron X-ray diffraction (0.6199 Å) analysis of the synthesized hydrides. **a** Peaks indexing for the La–Ce–H samples in DACs **#2,** **3** and the La–H sample in DAC **#S**. Insets show the integrated diffraction patterns. The wide diffraction band of an impurity located on the seat surface in DAC **#3** is masked by gridlines (Supplementary Fig. S10). **b** Pressure dependence of the unit cell volume of different polyhydrides. The experimental results for the La–Ce–H and La–H systems are shown in color and black, respectively. Gray symbols show literature data for the synthesized La–H phases^7,9,29^. Solid and dashed lines indicate the *P*–*V* relation of La–H and Ce–H phases, respectively. “VASP” marks the equation of state (EoS) calculated using the VASP code (PBE GGA), and “QE” marks the EoS calculated using the Quantum ESPRESSO code (PAW PBE).

To reveal the crystal structures of the superconducting polyhydrides, we performed synchrotron X-ray diffraction (XRD) measurements on the electrically characterized La–Ce–H samples in DACs **#2** and **#3** (Fig. 2a and Supplementary Figs. S4, S5, and S9), and newly prepared La–H samples in DAC **#S** (Fig. 2a and Supplementary Fig. S35). The data were collected from three individual La–Ce–H electrical DACs at their pressures and from La–H DAC **#S** during decompression. XRD reflections in phase mixtures were separated according to the phase distribution and the state of Debye rings. The *P*6_3_/*mmc* and *Fm*$\bar{3}$*m* structures were discovered in DAC **#3** at 131 GPa simultaneously, which can explain the high-*T*_c_ phases. The Debye rings of the *P*6_3_/*mmc* structure were spot-like in DAC **#2** while uniform in DAC **#3**. The lower pressure of synthesis and sufficient laser heating probably caused a better crystallization of sample **#2**. Noteworthy, the temperature (<1500 K) is not high enough to melt the La–Ce alloy at megabar pressures. Laser-heating may decompose the synthesized hydrides but cannot change the disordered distribution of La/Ce atoms. During the revision of our manuscript, we noticed another two La–Ce–H works with La/Ce of 1:1 that reported the *P*6_3_/*mmc*^36^ and *Fm*$\bar{3}$*m*^37^ phases, respectively. The main differences between our and their data lie in the different La–Ce ratios and synthesized conditions, which contribute to the various results. By comparing with *T*_c_–*P* trend (Fig. 4c and Fig. S36), we propose that the *Fm*$\bar{3}$*m* phase can possibly only be synthesized at pressures above 130 GPa (DACs **#3** and **#6**) with lower *T*_c_ than the *P*6_3_/*mmc* phase (Supplementary Figs. S8 and S18). Besides, the impurity phases with lower hydrogen content can explain the other lower *T*_c_s that we also observed. Current experimental techniques allow one just to determine the metal sublattice and estimate the hydrogen content in metal polyhydrides. Hydrogen atoms cannot be directly determined due to their very low scattering factor. Neutron diffraction^38^ and nuclear magnetic resonance^39–42^ could be employed in the future to give more information on the structure of H-sublattice.

We plotted our *P*–*V* data together with the calculated or experimentally reported equations of state (EoS) of binary Ce–H and La–H hydrides for comparison (Fig. 2b). The hydrogen content was determined by comparing the unit cell volume. We concluded that the main superconducting phase in La–Ce–H system below 130 GPa was deemed as *P*6_3_/*mmc*-(La,Ce)H_9-10_. Considering that the electrical resistance and XRD measurements are not performed on the same DAC for the binary La–H system, *T*_c_ values observed in DAC **#L1** cannot be directly distinguished from those of the mixed phases *P*6_3_/*mmc*-LaH_*x*_, and *C*2/*m*-LaH_*x*_ and *I*4/*mmm*-LaH_*x*_ in DAC **#S**. However, this does not affect the conclusion that ternary hexagonal La–Ce–H system exhibits higher *T*_c_ than the binary La–H system, both of which are synthesized at the same pressure-temperature conditions.

### The upper critical magnetic field

**Fig. 3:**
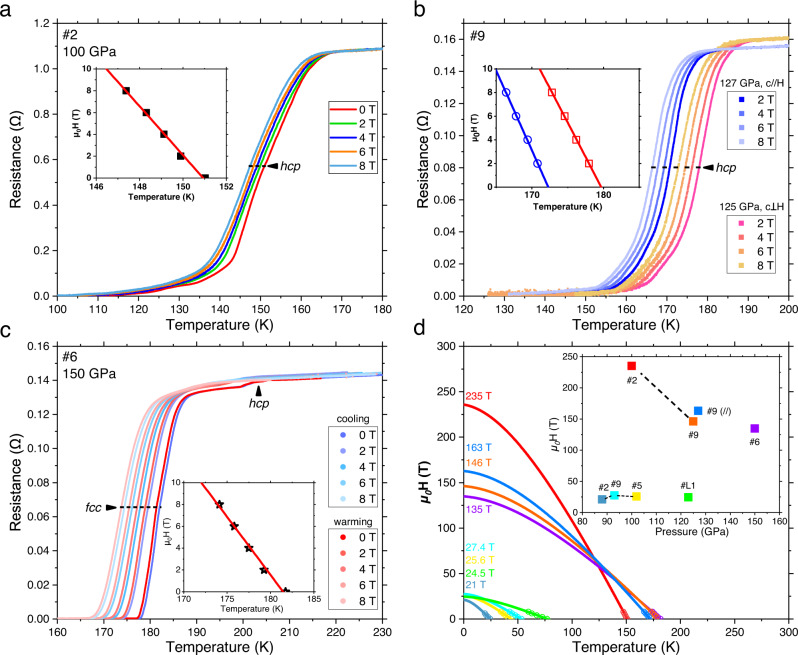
Electrical measurements of the superconducting transition in external magnetic fields. **a**–**c** Temperature dependences of the electrical resistance in external magnetic fields for different runs. The *T*_c_s are marked at the temperature in the middle of transition. Insets are the enlarged part of the fitting results using the simplified WHH formula^44^. **d** Extrapolated upper critical magnetic fields from different runs for La–Ce–H and La–H systems. Inset shows the obtained upper critical field *H*_c2_(0) at different pressures.

To further confirm the superconductivity and study the upper critical field *H*_c2_(0) of the synthesized superconducting phases, we have applied an external magnetic field to different DACs from 150 GPa to 88 GPa (Fig. 3). We used the *T*_c-mid_ that can be easily recognized to fit with the Werthamer-Helfand-Hohenberg (WHH) model^43^, simplified by Baumgartner^44^. For all the DACs, the *T*_*c*_ decreases with increasing magnetic field, as in all superconductors. The acceptable difference between the cooling and warming cycle is because of the temperature gradient between the temperature sensor and the target sample (Fig. 3c). We tried to apply the field parallel and perpendicular to the culet of DAC **#9** and observed the trace of anisotropy effect (Fig. 3b). During the decompression of the La–Ce–H system, the upper critical field at 0 K (obtained by extrapolation using the simplified WHH model) increased from 135 T (150 GPa) to striking 235 T (100 GPa), accompanied by a broadening of the transition width. After the decomposition of (La,Ce)H_9-10_, the *H*_c2_(0) for the residual phases dropped to ~25 T at 88–102 GPa and became steady in this pressure range. The La–H system shows a similar *H*_c2_(0) of 24.5 T at 123 GPa (Fig. S29). The strong enhancement of the upper critical magnetic field in the La–Ce–H system is probably related to the random distribution of La/Ce atoms and local distortion of the H-sublattice induced by it. This situation dramatically shortens the electronic mean free path and pushes the system into the dirty limit (Fig. S38). The H-sublattice then becomes extremely unstable and distorted near the decomposition pressure of the polyhydride.

## Discussion

**Fig. 4:**
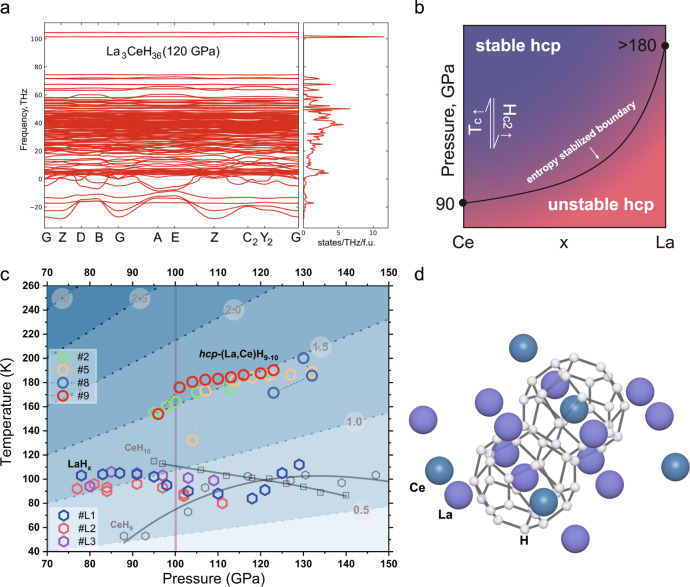
The stability and superconductivity of La–Ce–H system. **a** Phonon band structure and density of states of ordered *hcp*-La_3_CeH_36_ at 120 GPa calculated within the harmonic approximation. **b** Qualitative phase diagram of (La,Ce)H_9_ depends on the concentration and pressure. **c** *T*_c_–*P* relationship of the binary La–H and Ce–H, and ternary *P*6_3_/*mmc*-(La,Ce)H_9-10_. The gray dashed lines indicate the equivalent *S* values, which are marked in the white circles^61^. **d** The structure model of the *P*6_3_/*mmc*-(La,Ce)H_9-10_.

We further analyzed the dependence of superconducting state parameters by fitting the experimental *R*(*T*) data (see Supplementary Information for analysis). The fitted ${\theta }_{{{{{{\rm{D}}}}}}}$ and ω_log_ remain around 700–900 K at 90–150 GPa, suggesting the need of relatively high *λ* = 2–3 to ensure that the observed *T*_c_ is above 160 K. Rather high *λ* corresponds to the weakly ordered, soft, and highly defective structure of this superconducting La–Ce–H phase. The high-*T*_c_ (La,Ce)H_9-10_ can be preserved down to about 100 GPa and becomes dynamically unstable at lower pressures. However, we cannot rule out the possibility that (La,Ce)H_9-10_ is metastable in our studied pressure range, considering that both LaH_9-10_ and CeH_9-10_ phases are not dynamically and thermodynamically stable at pressures below 150 GPa according to the predicted convex hull^6,30^. The configurational entropy may contribute significantly to the stability at high temperatures because of the disordered state and laser heating to over a thousand kelvins^45^. Since it is hard to provide direct evidence on the influence of configurational entropy in the experiment, we have resorted to theoretical calculations. We calculated the dynamical stability (Fig. 4a and Supplementary Fig. S42) and superconducting properties of periodic structure (Supplementary Fig. S40 and Table S4) with different Ce content at 120 GPa with the harmonic approximation. All hexagonal LaCeH_18_, La_3_CeH_36_, and LaH_9_ phases showed imaginary phonon frequencies, while adding Ce decreased the extent of this instability. This indicates that adding Ce is beneficial for stabilizing this phase. At the same time, we should also consider the effects of anharmonicity and configurational entropy on the stabilization (Fig. 4b). In order to calculate the harmonic Eliashberg functions, soft modes <5 THz were excluded. We obtained a relatively smooth Eliashberg function of La_3_CeH_36_ similar to what was reported in the disordered or amorphous superconductors before^46^. However, the calculated *T*_c_ (131 K, Supplementary Fig. S40c) is about 50 K lower than the experimental value (185 K) at 120 GPa. The random mixing may non-trivially affect the structure, chemical bonding, and electronic properties^47^. Currently, theoretical calculations of La–Ce–H cannot provide an accurate prediction for this disordered system. The problem is in the computational complexity of calculations of ternary systems with variable element concentrations appearing due to the need to use unit cells with a large number of atoms (>40) and limited density of k and q-meshes. The synthesized *P*6_3_/*mmc*-(La,Ce)H_9-10_ can be viewed as a high-entropy superconducting alloy composed of a distorted H-sublattice and randomly substituted metal sublattice analogous to the high-entropy ceramics^48^ (Fig. 4d). Based on this, the La–Ce alloy can be replaced by various related high-entropy reactants to further improve the stability and superconducting properties.

In conclusion, we successfully synthesized the HTSC ternary La–Ce–H (La: Ce = 2.5~3.5) and binary La–H compounds at pressures lower than 130 GPa. *T*_c_ of *hcp*-(La,Ce)H_9-10_ was preserved to 176 K at about 100 GPa, which meant a high figure of merit (*S* = 1.62) comparable to *Fm*$\bar{3}$*m*-LaH_10_. Besides, the extrapolation of the upper critical magnetic field gives *H*_c2_(0) = 235 T at 100 GPa, the highest value for polyhydrides obtained so far by fitting with the simplified WHH formula. For comparison, *T*_c_ of the binary La–H phase was detected as 112 K at 129 GPa, decreased to 84 K at 118 GPa, and then increased to above 100 K at 78 GPa. Combining the analysis of experimental data and theoretical calculations, we think that the disordered state in the La–Ce–H system contributes to the giant enhancement of the superconducting *T*_c_ and *H*_c2_(0) compared with binary La–H and Ce–H systems. Importantly, we propose a new strategy for searching for HTSC hydrides at moderate pressure that uses multiple appropriate elements to maximize the configurational entropy of clathrate structures.

## Methods

### Experimental details

The La–Ce alloys were prepared using the multitarget magnetron sputtering (Supplementary Fig. S1). We sputtered the La (99.9%) and Ce (99.9%) metals simultaneously to the glass slide using DC and RF power supplies, respectively. The Ar pressure was 1.5 Pa, and both targets were pre-sputtered to remove the surface oxides. The La–Ce alloy was further characterized using the scanning electron microscope (SEM) Regulus 8100, equipped with the energy-dispersive X-ray spectroscopy (EDX). The La–Ce alloys for high-pressure experiment were located on the glass side very close to the SEM-characterized area and kept strictly in the glove box (O_2_ < 0.01 ppm, H_2_O < 0.01 ppm).

Normal type-Ia diamonds with 60–150 μm culets single-beveled to 250–300 μm were used; the pressure was measured according to the Raman vibration edge of diamond using Akahama’s calibration^49,50^. To protect the electrodes during decompression, we tested the nano-polycrystalline diamonds (NPD) without bevels in some runs (Supplementary Fig. S31). Because of the high fluorescence background of the NPDs, their Raman edge cannot be distinguished with 532 nm laser. Therefore, the NPDs (200–300 µm culet) were combined with the normal diamonds, and the electrodes were set on the NPD side. The indentation of a tungsten gasket was insulated using c-BN/epoxy first, and the bevel part was filled with oxides (Al_2_O_3_, MgO)/epoxy. The electrodes were integrated to the diamond by a lithographic Mo (300–500 nm thick)^51^ or manually cutting Pt (2–3 µm thick) foil. The La–Ce samples were loaded into the chamber filled with ammonia borane (AB), which acted as a hydrogen source. The sample was heated at the target pressure by a 1070 nm infrared laser with 3–5 µm focus spot and an exposure time of 1–3 s. Afterward, we put the DAC into a helium cryostat (1.5–300 K) equipped with a 0–9 T superconducting magnet for low-temperature electrical measurements. The resistance was measured using the four-probe method with the delta model of the Keithley current source (Model 6221, 1 mA) and voltmeter (Model 2182A). The crystal structure was determined using the synchrotron X-ray diffraction (XRD) on the BL15U1 synchrotron beamline with a wavelength of 0.6199 Å at the Shanghai Synchrotron Research Facility (SSRF). The experimental XRD images were integrated and analyzed for possible phases using the Dioptas software package^52^. To fit the diffraction patterns and obtain the cell parameter, we analyzed the data using Materials Studio and Jana2006 software^53^, employing the Le Bail method^54^.

### Theoretical calculations

The calculations of superconducting *T*_c_ were carried out using Quantum ESPRESSO (QE) package^55,56^. The phonon frequencies and electron–phonon coupling (EPC) coefficients were computed using the density functional perturbation theory^57^, employing the plane-wave pseudopotential method with a cutoff energy of 70–80 Ry, and the Perdew–Burke–Ernzerhof exchange–correlation functional^57–59^. Within the optimized tetrahedron method^60^, we calculated the electron–phonon coupling coefficients λ and the Eliashberg functions via sampling of the first Brillouin zone by 12 × 12 × 8 *k*-points and 3 × 3 × 2 *q*-points meshes. To evaluate the density of states (DOS) and electron–phonon linewidths, a denser 16 × 16 × 12 *k*-mesh was used. PBE PAW pseudopotentials for Ce, La, and H (pbe-spfn-kjpaw_psl) were used with a plane-wave basis set cutoff of 70 Ry for calculations of superconducting properties of (La,Ce)H_9_. The *k*-space integration (for electrons) was approximated by a summation over the 8 × 6 × 6 uniform grid in reciprocal space, and the used supercell was up to La_3_CeH_36_. Dynamical matrices and electron–phonon linewidths were calculated on a uniform 4 × 3 × 3 grid in *q*-space. The harmonic approximation was used, therefore, the obtained electron–phonon coupling coefficients for lanthanum and lanthanum-cerium polyhydrides at low pressures may be overestimated.

## Supplementary information

Supplementary Information

## Data Availability

The authors declare that the main data supporting our findings of this study are contained within the paper and Supplementary Information. Because the current database contains many other miscellaneous items, we cannot sort them out in a short time. All other relevant data are available from the corresponding author upon request.

## Code availability

USPEX code is free for academic use and available after registration at https://uspex-team.org/en. Quantum ESPRESSO code is free for academic use and available after registration at https://www.quantum-espresso.org/. VASP code is available for download on the developer page: https://www.vasp.at/. The processing of the Quantum ESPRESSO calculation was carried out using the code available on GitHub: https://github.com/GitGreg228/a2f.
