# Supplementary material for: Enhancement of superconducting properties in the La–Ce–H system at moderate pressures
Source: Nat Commun. 2023 May 9;14:2660. doi: 10.1038/s41467-023-38254-6 (PMC10170082; doi:10.1038/s41467-023-38254-6)
Supplement: Supplementary file 1 — Supplementary Information [file 41467_2023_38254_MOESM1_ESM.pdf]

Supplementary Information

Enhancement of superconducting properties in the La–Ce–H system at moderate pressures

Wuhao Chen[1], Xiaoli Huang[1,*], Dmitrii V. Semenok[2], Su Chen[1], Di Zhou[2], Kexin Zhang[1], Artem R. Oganov[3], and Tian Cui[1,4*]

[1] State Key Laboratory of Superhard Materials, College of Physics, Jilin University, Changchun 130012, China
[2] Center for High Pressure Science and Technology Advanced Research (HPSTAR), Beijing 100094, China
[3] Skolkovo Institute of Science and Technology, Skolkovo Innovation Center, Bolshoy Boulevard 30, bldg. 1, Moscow 121205, Russia
[4] School of Physical Science and Technology, Ningbo University, Ningbo 315211, China

*Corresponding authors' e-mails: huangxiaoli@jlu.edu.cn, cuitian@nbu.edu.cn

Summary of all Runs

Table S1. The experimental details for different experimental runs. The La: Ce ratios of the alloys in runs #1,2,3 and 7 should be close to 3, because these alloys were prepared in similar conditions (as shown in the below Fig. S1).

Run	Initial sample	La:Ce	Pressure before and after laser heating	Measurements
#1	(La,Ce)+AB	Unknown	102–82 GPa	XRD
#2	(La,Ce)+AB	Unknown	113–112 GPa	XRD, R–T
#3	(La,Ce)+AB	Unknown	130–131 GPa	XRD, R–T
#4	(La,Ce)+AB	≈6	107–103 GPa	EDX, R–T
#5	(La,Ce)+AB	3.2–3.5	129–132 GPa	EDX, R–T
#6	(La,Ce)+AB	2.7–2.9	152–150 GPa	EDX, R–T
#7	(La,Ce)+AB	Unknown	without laser-heating	R–T
#8	(La,Ce)+AB	≈3	129–131 GPa, 131–130 GPa	EDX, R–T
#9	(La,Ce)+AB	3.4–3.5	120–118 GPa, 122–120 GPa, 127–125 GPa	EDX, R–T
#L1	La+AB	Pure La	125–123 GPa, 130–127 GPa, 132–129 GPa	R–T
#L2	La+AB	Pure La	113–111 GPa	R–T
#L3	La+AB	Pure La	109 GPa heated	R–T
#S	La+AB	Pure La	132–130 GPa	XRD

Alloy Synthesis

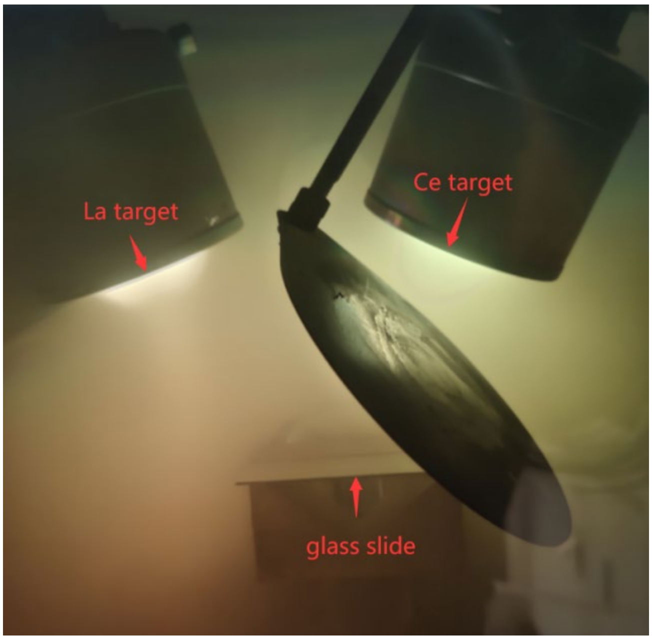

Fig. S1. Photograph of the chamber inside the magnetron sputtering equipment. The La and Ce ions were mixed in the Ar atmosphere and then deposited on a glass slide.

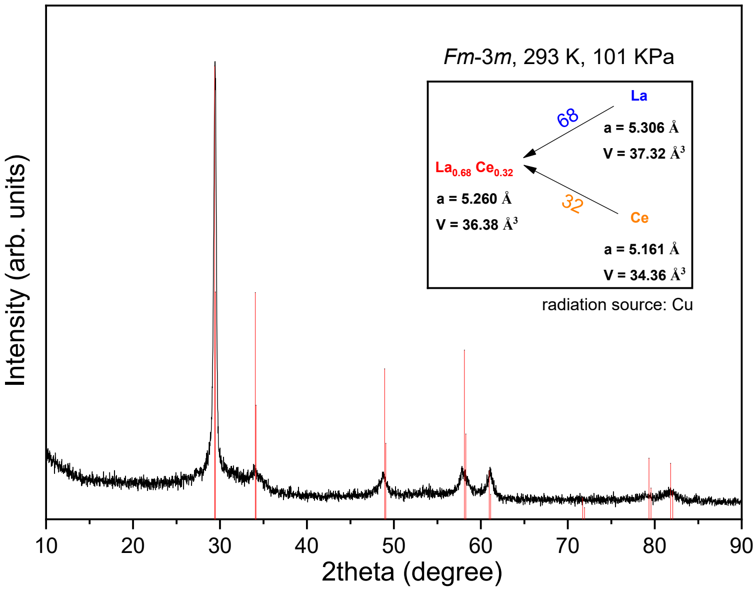

Fig. S2. XRD (Cu: $K_{\alpha1}$=1.54056Å, $K_{\alpha2}$=1.54439Å) of a typically prepared La–Ce alloy at ambient conditions. Red lines show the indexing results of the calculated diffraction of $Fm\bar{3}m$ structure. The related cell parameters are shown in the inset. The La:Ce ratio was estimated according to the calculation of cell volume. However, the La contents decreases to 64% according to the reported *a*-spacing of La-Ce alloy because of the deviation from the linear relation[1].

Run #1

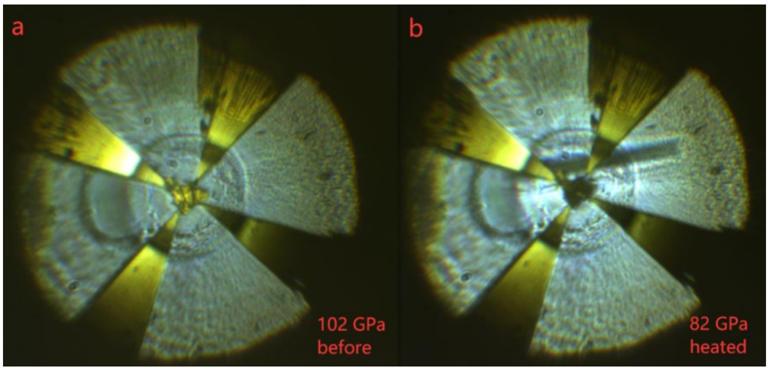

Fig. S3. Photographs of DAC #1 (a) before and (b) after laser-heating. A vertical crack appeared and grew gradually during the laser-heating.

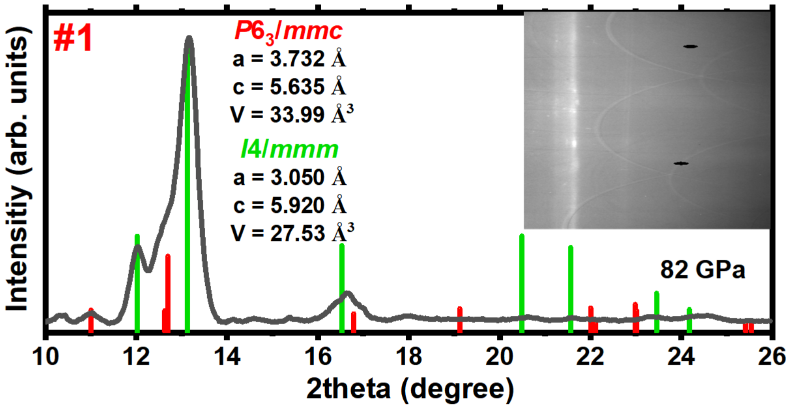

Fig. S4. Le Bail fitting of the XRD pattern of the sample in DAC #1 at 82 GPa. The inset shows the integrated XRD pattern.

Run #2

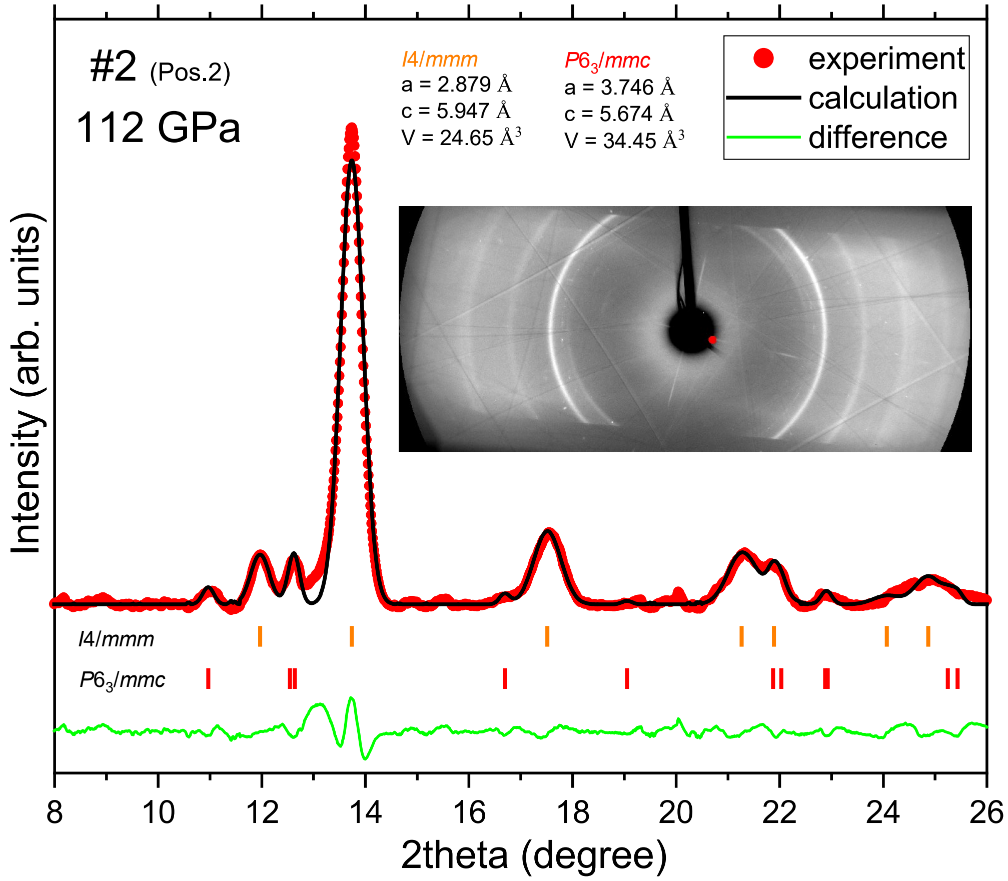

Fig. S5. Le Bail fitting of the XRD pattern for another detected position of DAC #2 at 112 GPa. The inset shows the XRD pattern.

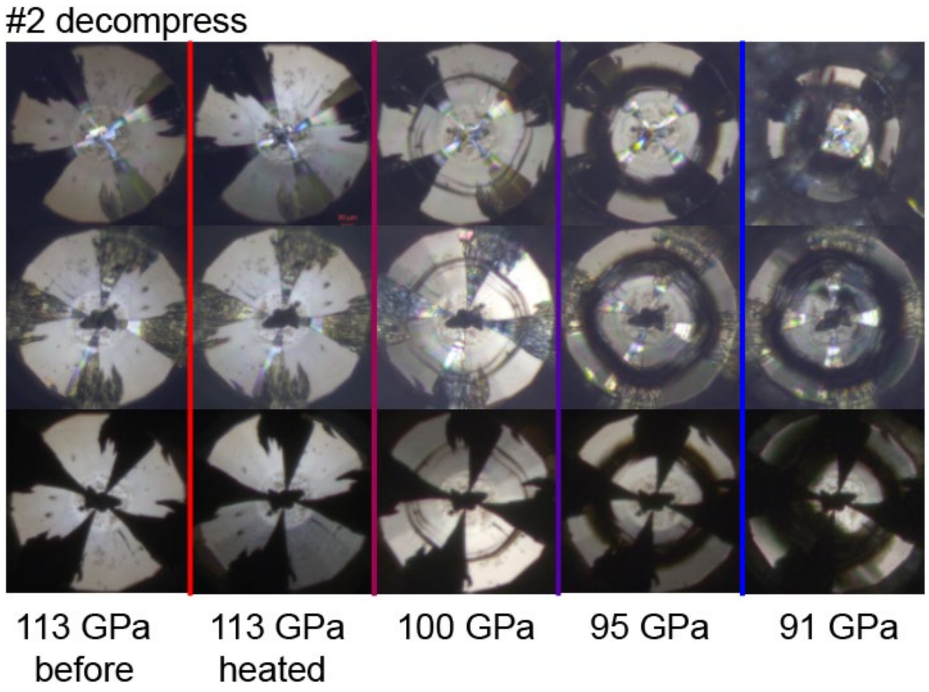

Fig. S6. Changes in the diamond's bevel during the decompression of DAC #2 from different photographic views.

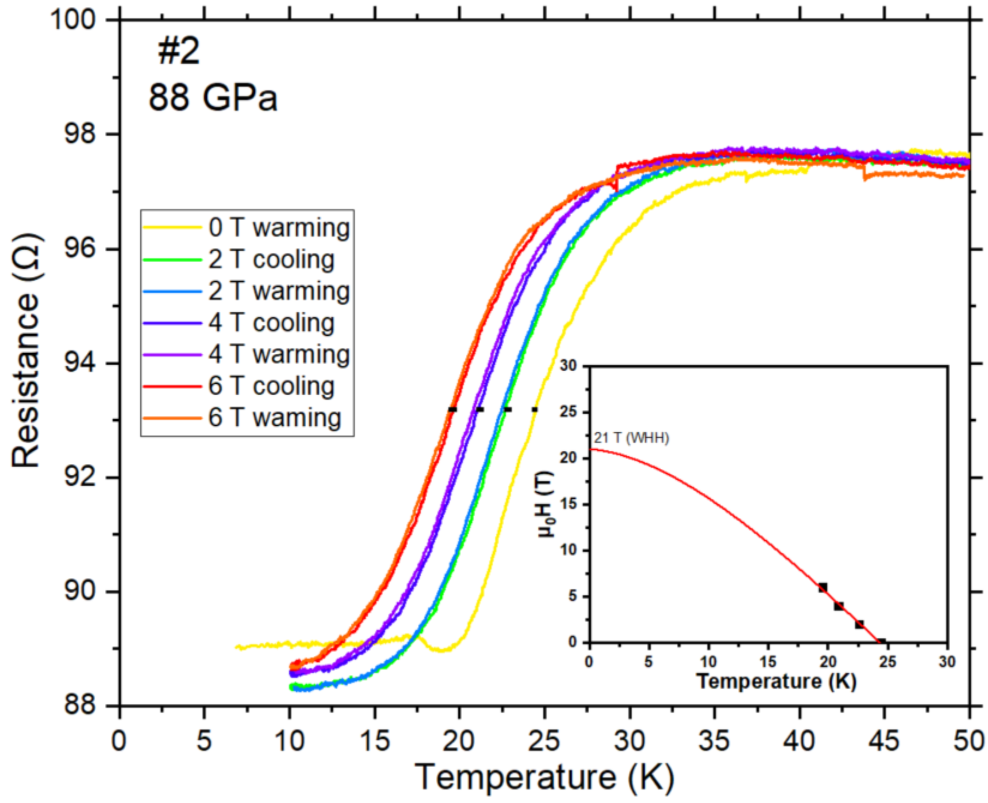

Fig. S7. Superconducting transitions of sample #2 under an external magnetic field at 88 GPa. Black dots indicate the positions where the T_cs were taken. Inset is the fitting with simplified WHH formula.

Run #3

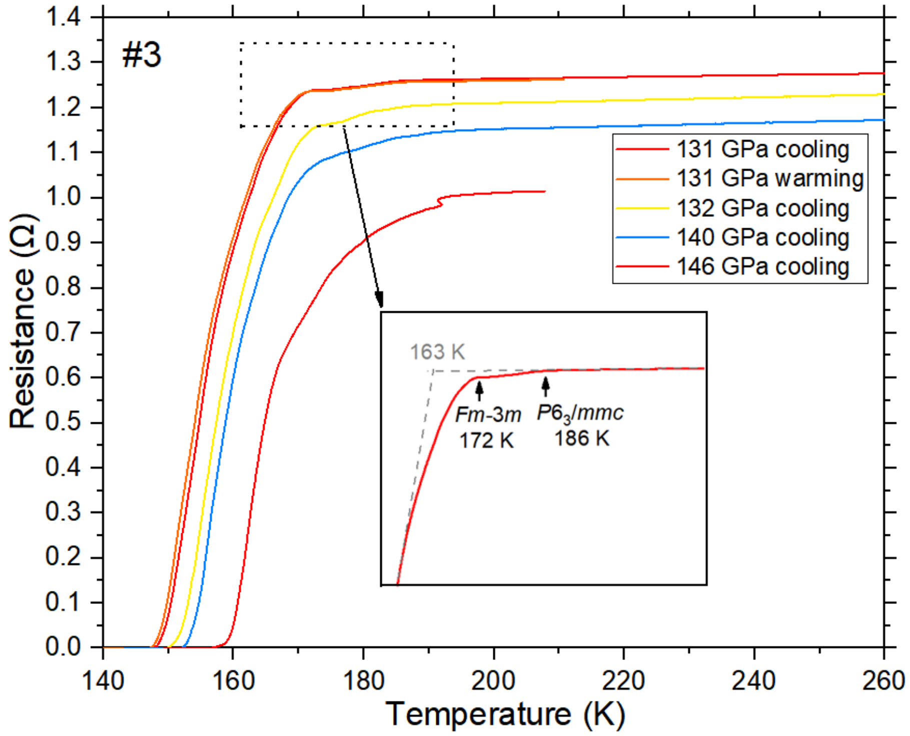

Fig. S8. Temperature dependence of the electrical resistance for the La-Ce-H sample in DAC #3 at different pressures. Inset is the enlarged curve of 131 GPa cooling.

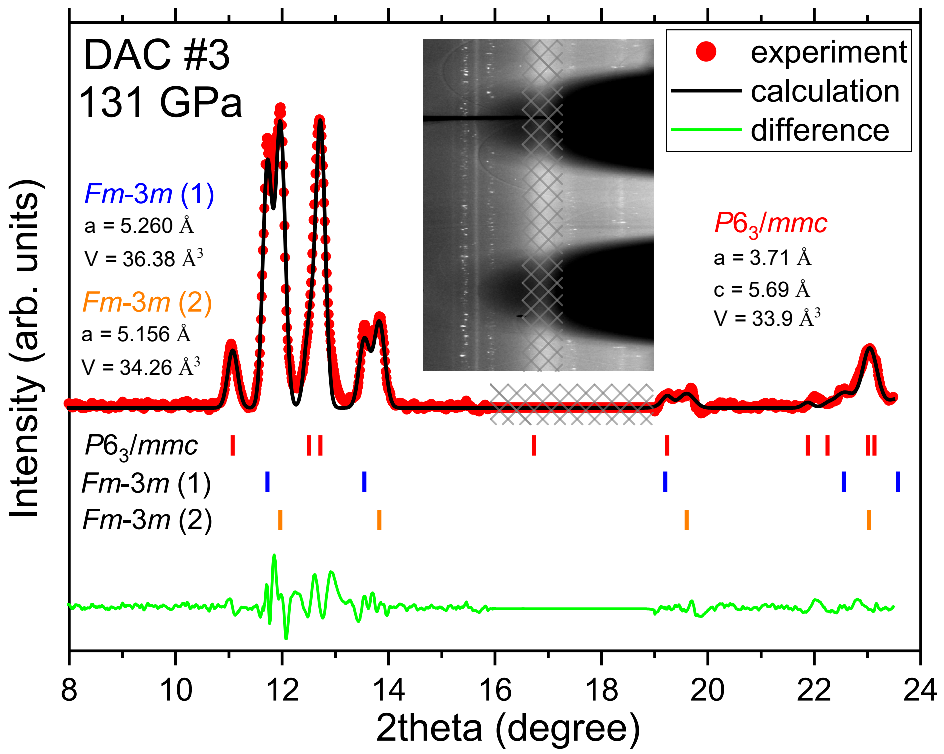

Fig. S9. Le Bail fitting of the XRD pattern of the sample in DAC #3 at 131 GPa. The inset shows the integrated diffraction pattern. The diffraction on the impurity that located on the seat surface of the diamond, as shown below, is masked by gridlines.

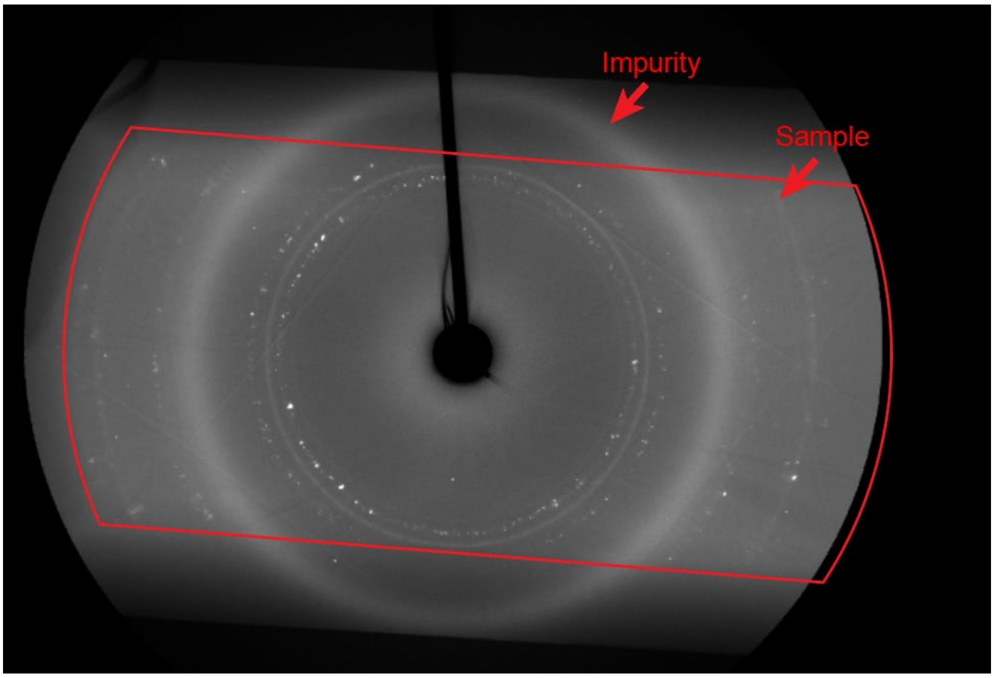

Fig. S10. X-ray diffraction pattern of the sample in DAC #3 at 131 GPa. The red frame represents the projection of the long opening angle in the diamond seat. The sample shows a set of incomplete diffraction circles because of the occlusion of the seat, whereas the impurity shows a full broad entire circle. This means the impurity is not in the sample chamber and is closer to the diamond seat (possibly some dirt on the seat surface of diamond).

Run #4

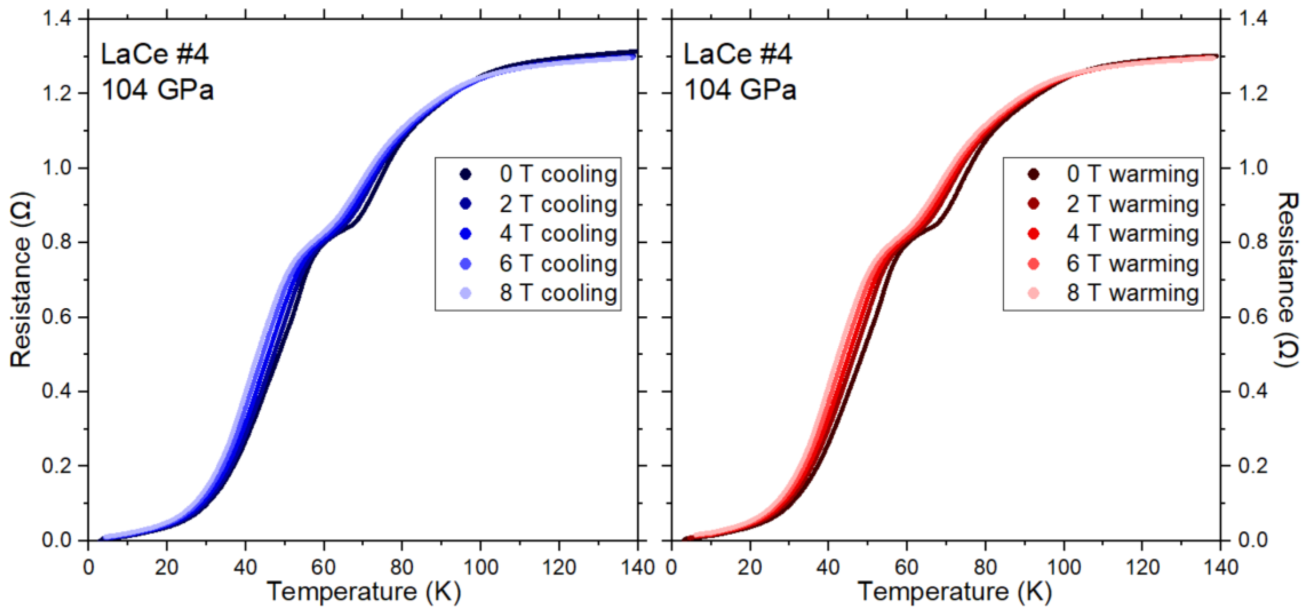

Fig. S11. Temperature dependence of the electrical resistance in DAC #4 near the superconducting transition in an external magnetic field for cooling(left) and warming(right) cycles.

Run #5

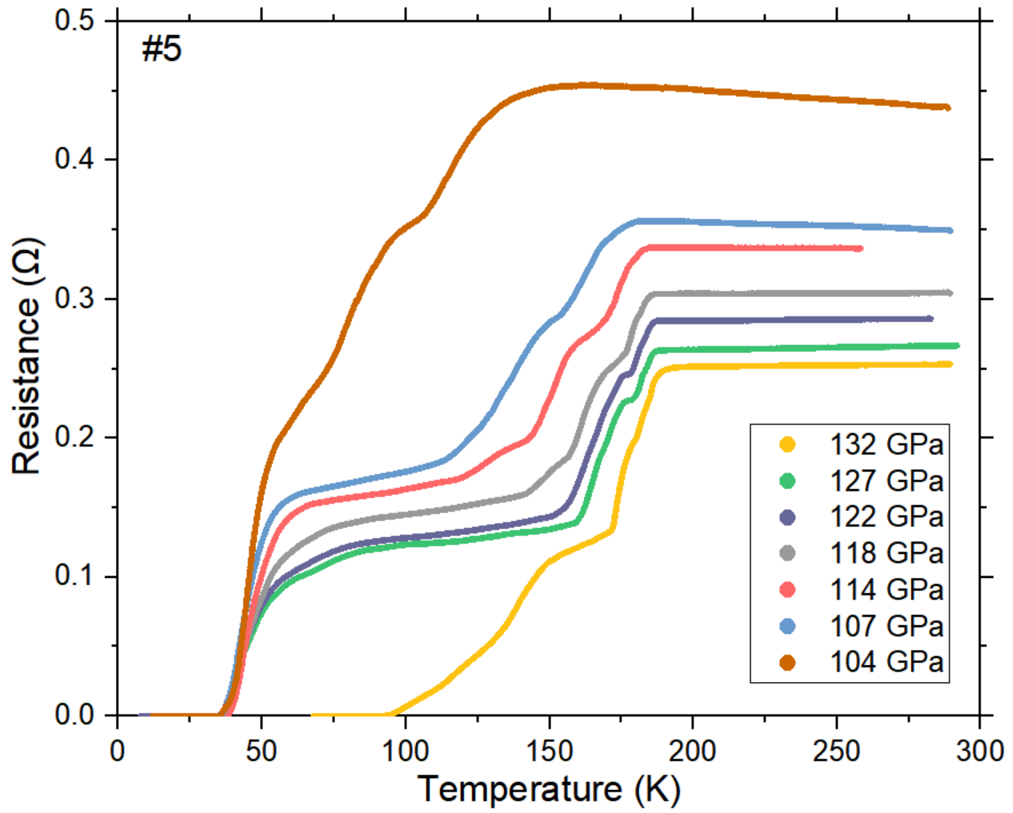

Fig. S12. Temperature dependence of the electrical resistance for the La-Ce-H sample in DAC #5 at different pressures.

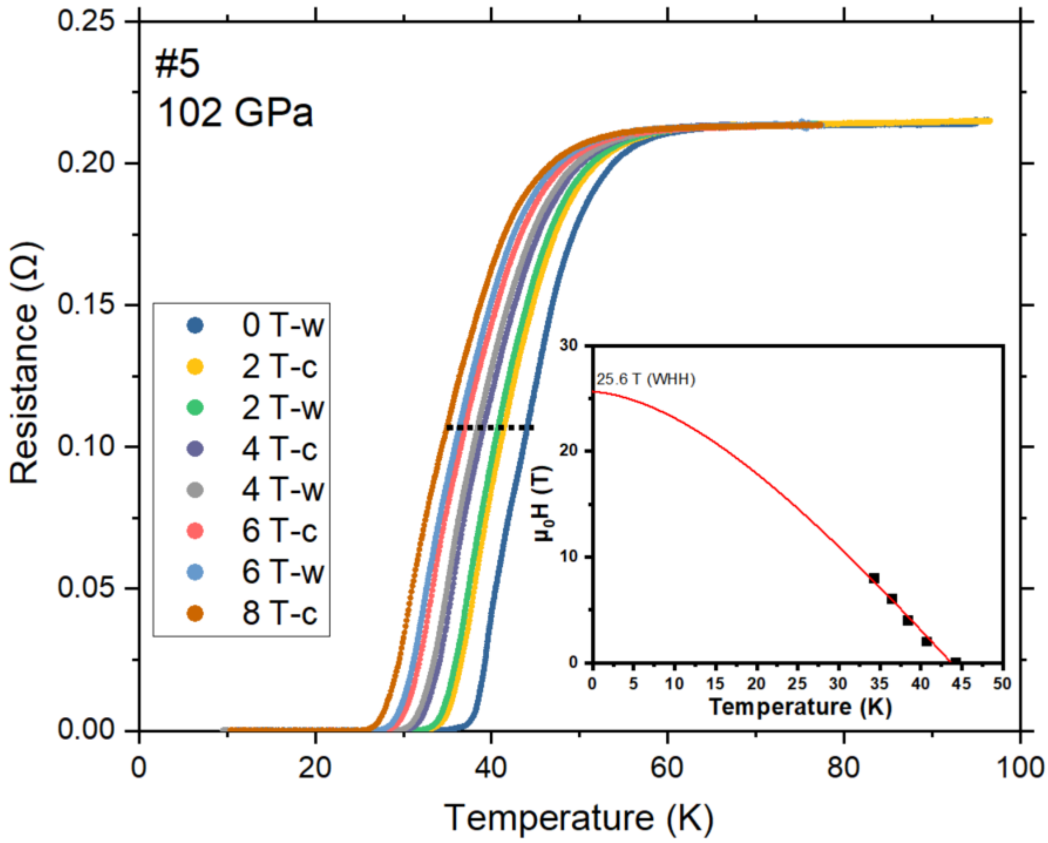

Fig. S13. Superconducting transitions of sample #5 under an external magnetic field at 102 GPa upon the cooling and warming process. Black dots indicate the positions where the T_cs are taken. Inset is the fitting with the simplified WHH formula.

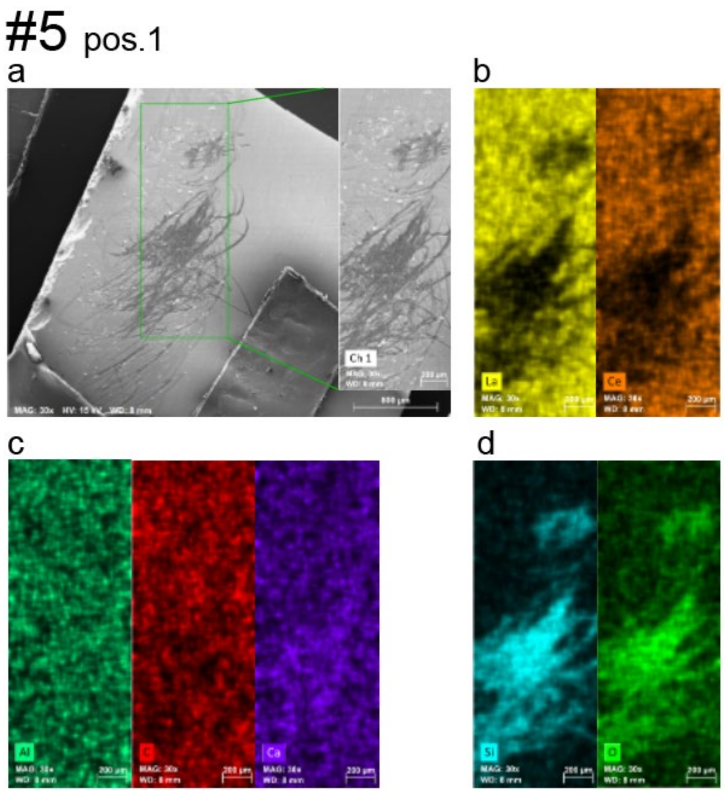

Fig. S14. Scanning electron microscopy (SEM) and energy dispersive X-ray spectroscopy (EDX) analysis of the La–Ce alloy in DAC #5, pos. 1 (a) The SEM photo of the sample on the glass slide. (b–d) Elements distribution in the rectangular area shown by green lines in panel (a).

In DAC #5, the alloy composition was characterized after the sample loading. We used a tungsten needle to scratch the La–Ce layer on the glass slide to get particles, which left traces (Fig. S14a). La and Ce were both uniformly distributed except for the scratched area (Fig. S14b). Si and O mainly come from the glass slide, thus the scratched area has high intensity (Fig. S14d). On the La–Ce surface, there was a tiny amount of oxygen because of the oxides formed during the transfer. Moreover, Al, C and Ca exist all over the selected region and can be viewed as a background signal. Fig. S16 shows that the scratched particles have a uniform distribution of La and Ce. The sputtered La–Ce can form clusters or smooth solid solutions.

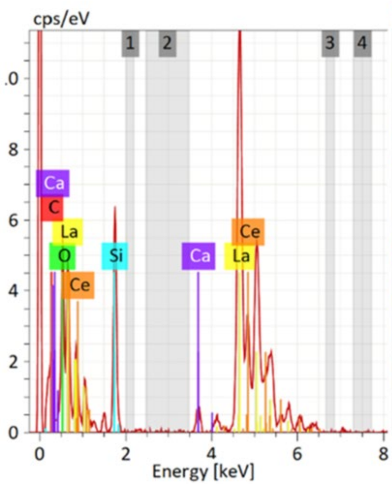

Map

Element	Line	Weight [%]	Atom [%]
O	K-Serie	7.65	28.40
Si	K-Serie	6.12	12.94
La	L-Serie	61.73	26.39
Ce	L-Serie	18.57	7.87
C	K-Serie	4.38	21.66
Ca	K-Serie	0.94	1.39
Al	K-Serie	0.61	1.34
		**100.00**	**100.00**

Fig. S15. Compositions analyzed using the EDX in DAC #5.

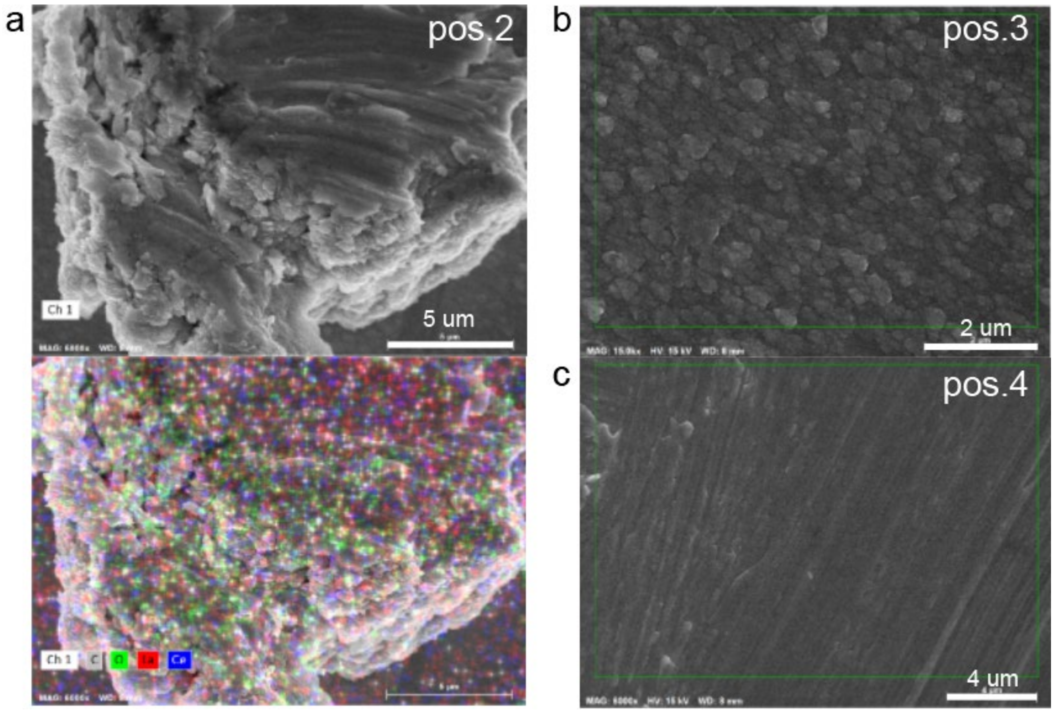

Fig. S16. The SEM photos of other positions in DAC #5. (a) The selected particle (top) and the element distribution on it (bottom). (b, c) Positions 3 and 4.

Run #6

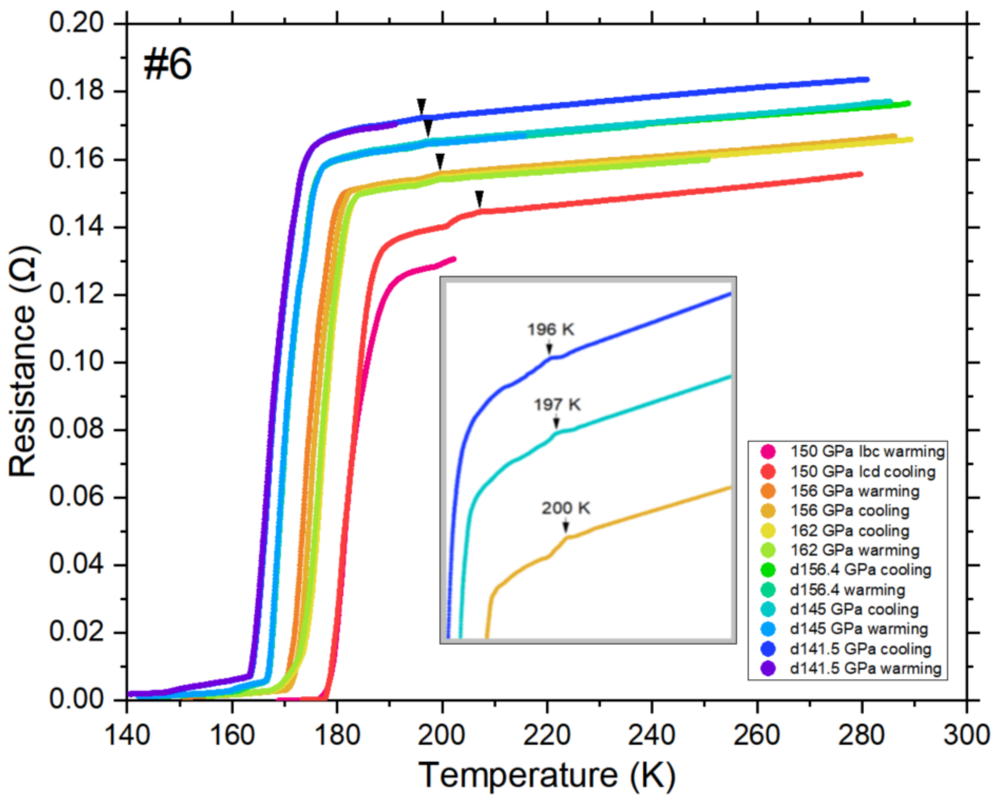

Fig. S17. Temperature dependence of the electrical resistance for the La-Ce-H sample in DAC #6 at different pressures. Inset shows the enlarged curves. The arrows indicate the T_c of *hcp* phase.

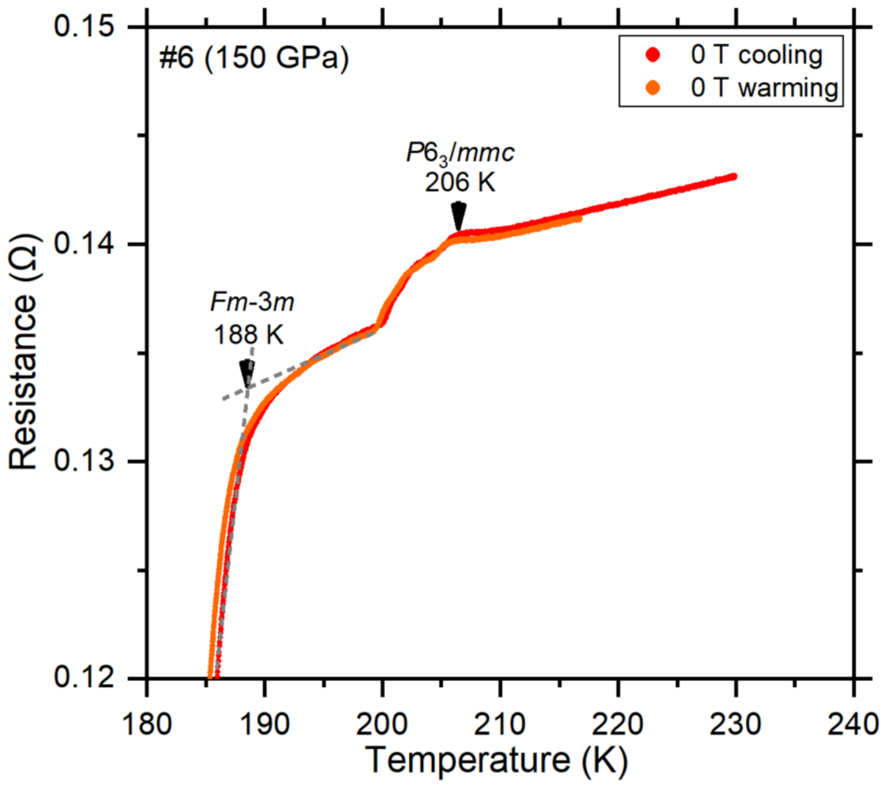

Fig. S18. Enlarged curves in Fig. 3 at 0 T with the marks of transition temperature.

Run #7

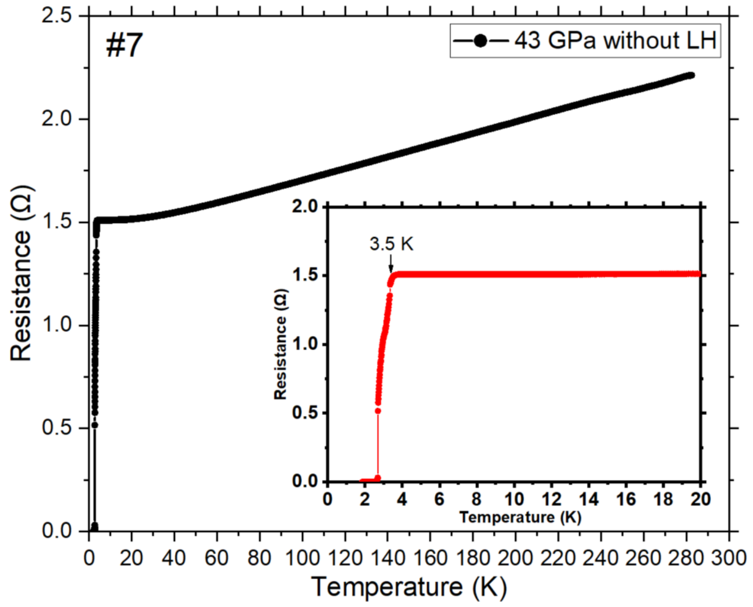

Fig. S19. Temperature dependence of the electrical resistance for La–Ce alloy in run #7 at 43 GPa before the laser-heating (LH). Inset shows the enlarged part of the plot.

Run #8

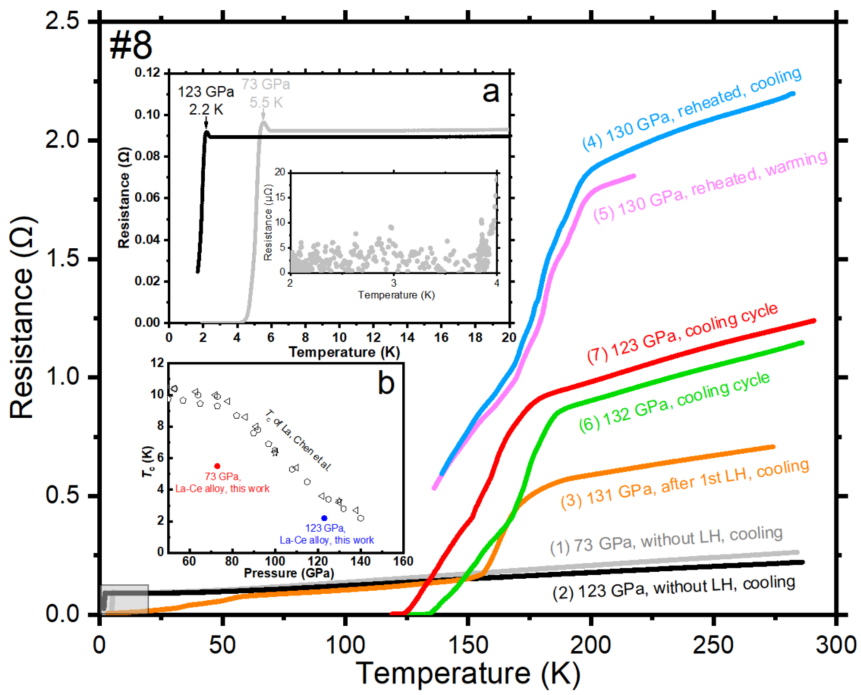

Fig. S20. Temperature dependence of the electrical resistance in DAC #8 at different conditions. Numbers from (1) to (7) indicate the sequence of the experiments. Inset (a) shows the enlarged part of the plot, with the superconducting transition of La–Ce alloy before the laser-heating. In inset (b), a comparison of T_c of the La–Ce alloy and pure La is shown[2].

Run #9

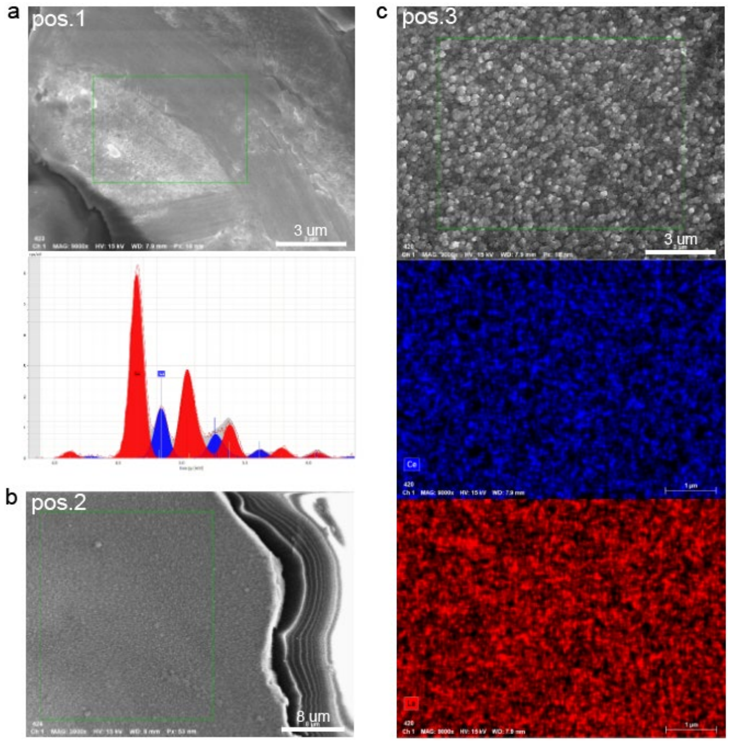

Fig. S21. The EDX analysis of the La–Ce alloy in DAC #9. (a) Top: the selected particle, bottom: L-Serie energy spectrum of the La–Ce alloy. (b) SEM photo at position 2. (c) Top: SEM photo at position 3, middle: Ce distribution, bottom: La distribution.

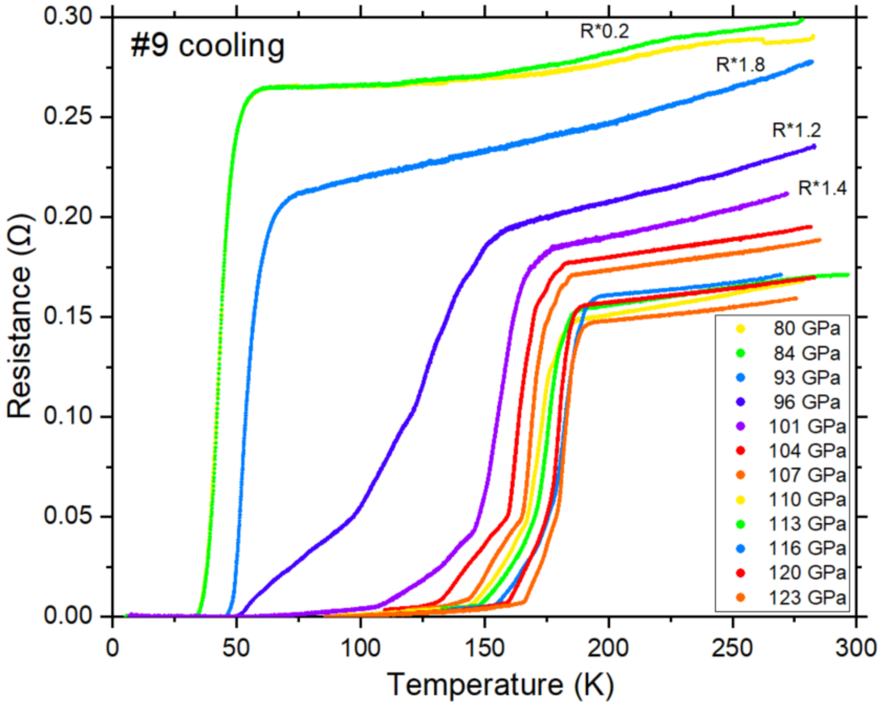

Fig. S22. Temperature dependence of the electrical resistance for the La-Ce-H sample in DAC #9 in the cooling cycle at different pressures. For the curves at low pressure, the resistances are multiplied by different factors.

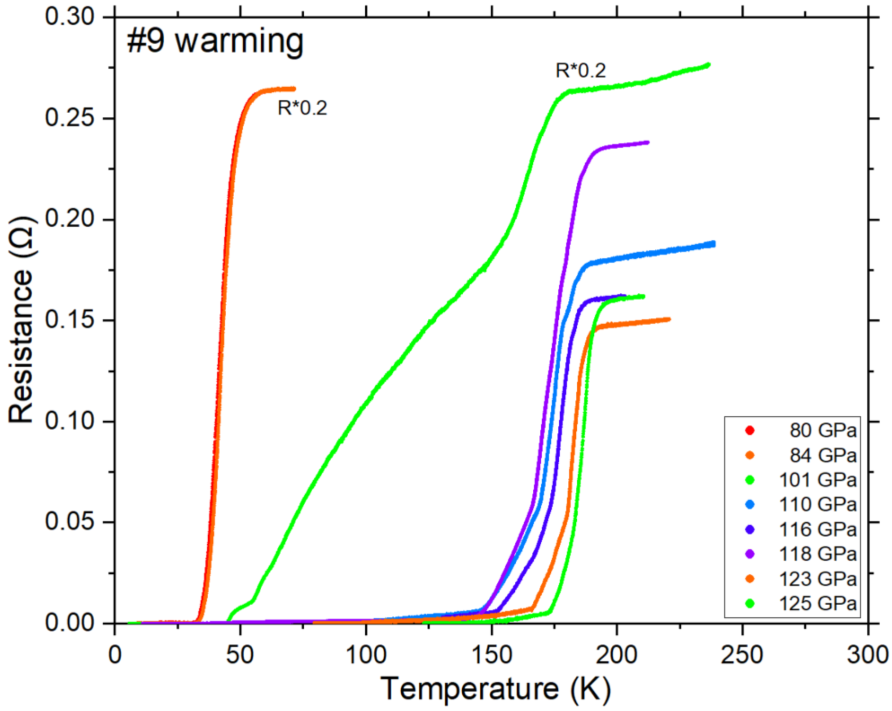

Fig. S23. Temperature dependence of the electrical resistance for the La-Ce-H sample in DAC #9 in the warming cycle at different pressures. For the curves at low pressure, the resistances are multiplied by factors 0.2.

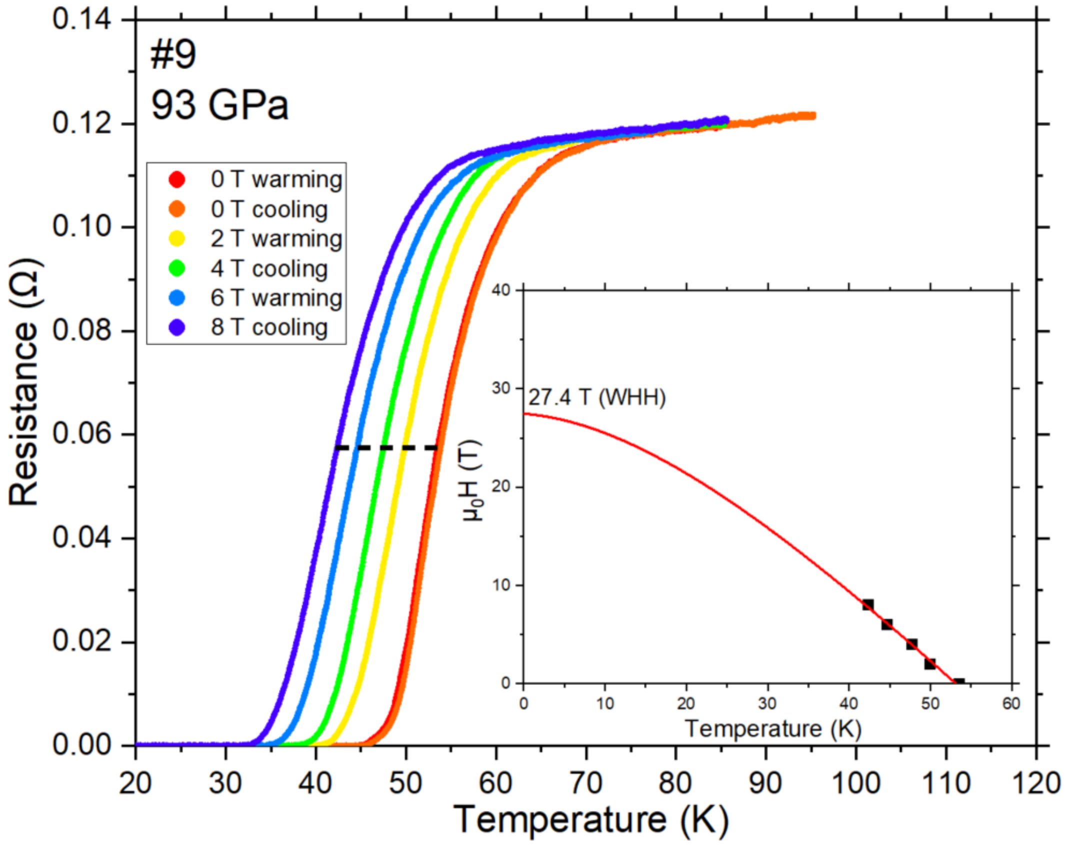

Fig. S24. Superconducting transitions of sample #9 under an external magnetic field at 93 GPa. Black dots indicate the positions where the T_cs where taken. Inset is the fitting with simplified WHH formula.

Run #L1

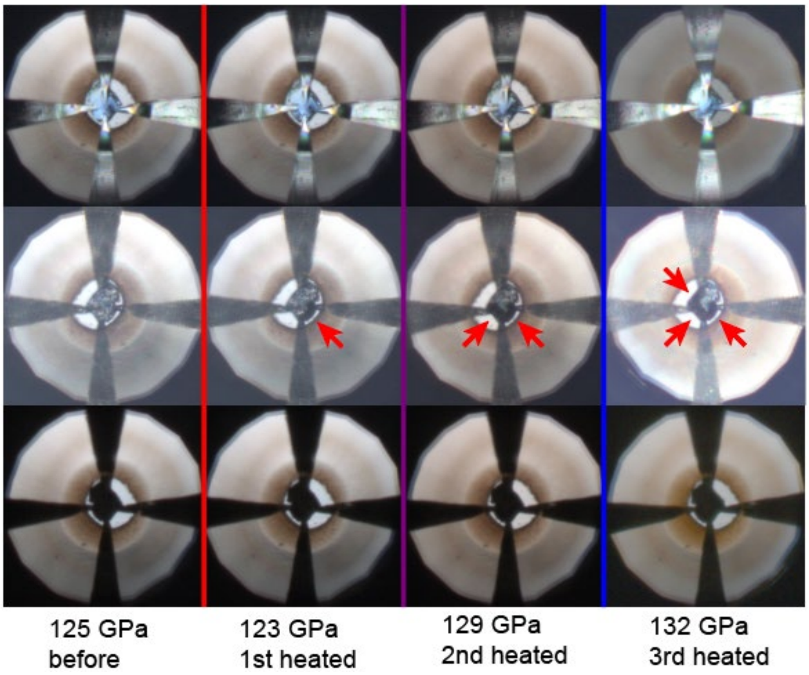

Fig. S25. Photographs of the sample in DAC #L1 before and after laser-heating for three times. The tips of four Pt electrodes were directly compressed to the edge of the sample. Arrows show the changes of the sample after the heating.

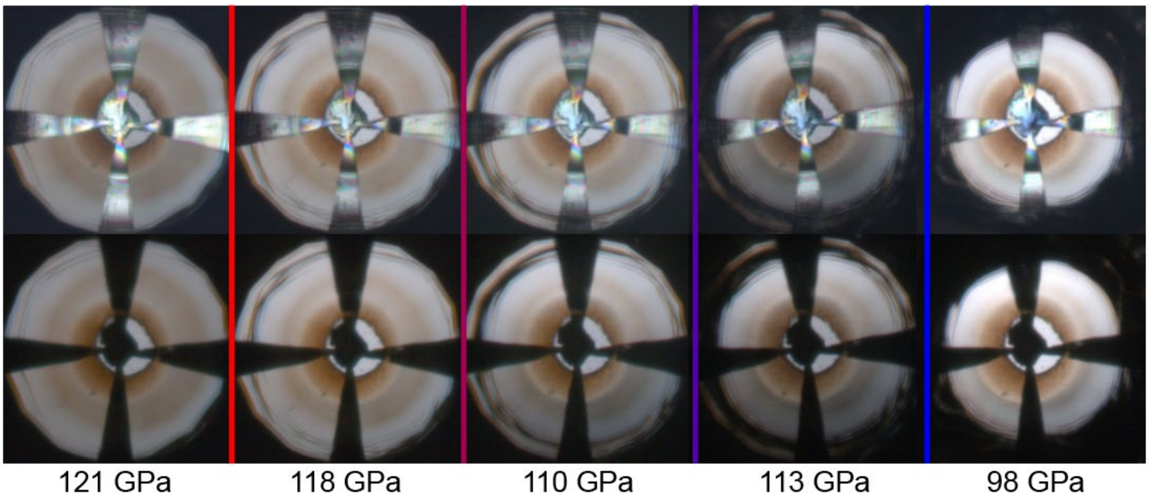

Fig. S26. Changes of the diamond's bevel during decompression of DAC #L1 at different pressures.

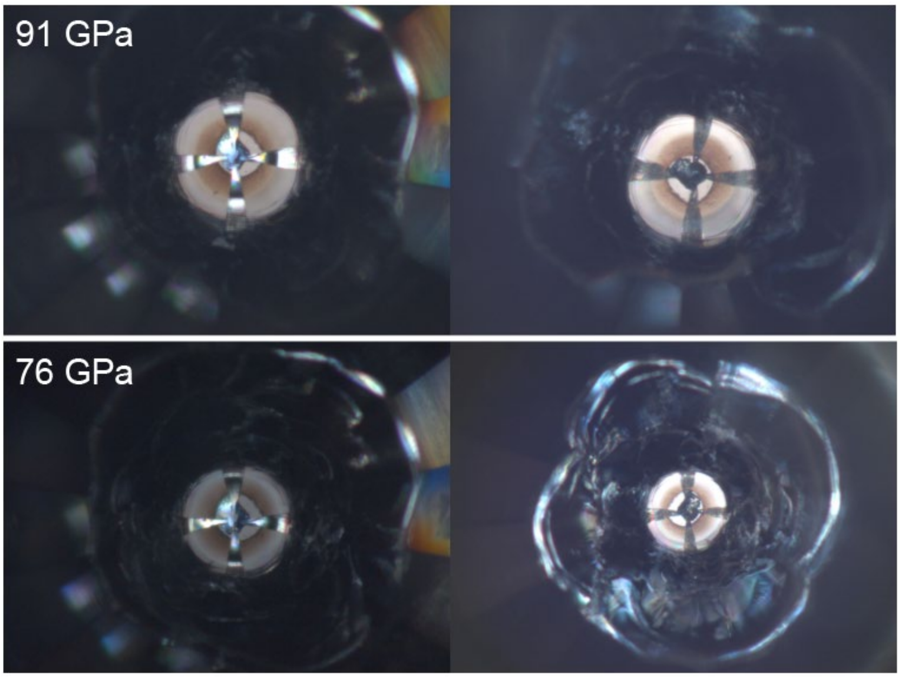

Fig. S27. Front and back views of the diamonds in DAC #L1 at 91 GPa and 76 GPa.

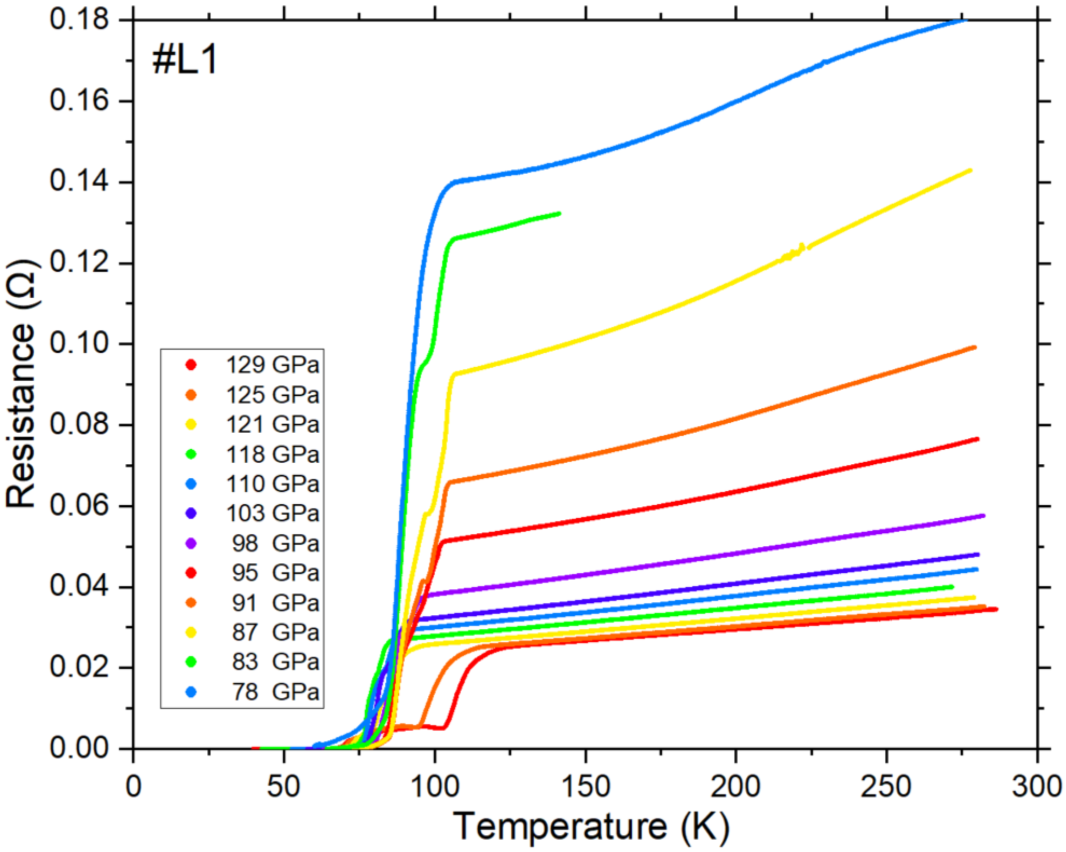

Fig. S28. Temperature dependence of the electrical resistance for the La- H sample in DAC #L1 at different pressures.

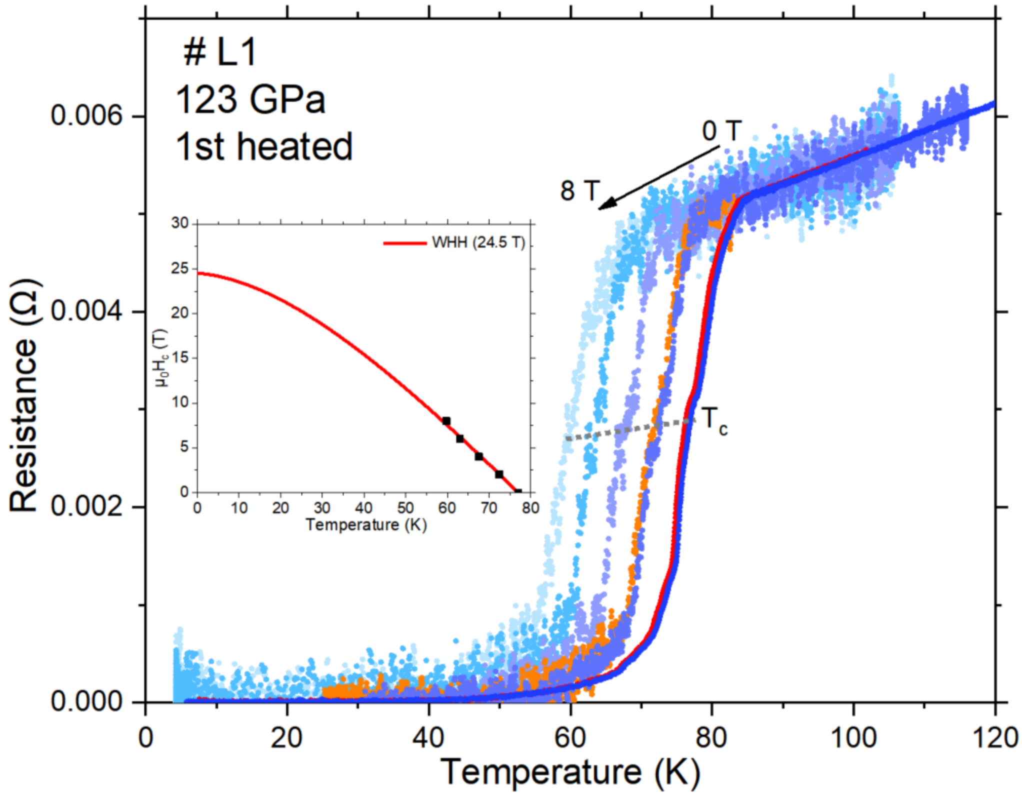

Fig. S29. Superconducting transition in DAC #L1 characterized by the temperature dependence of the resistance in an external magnetic field at 123 GPa after the first laser-heating. Inset shows the WHH fitting of the experimental data.

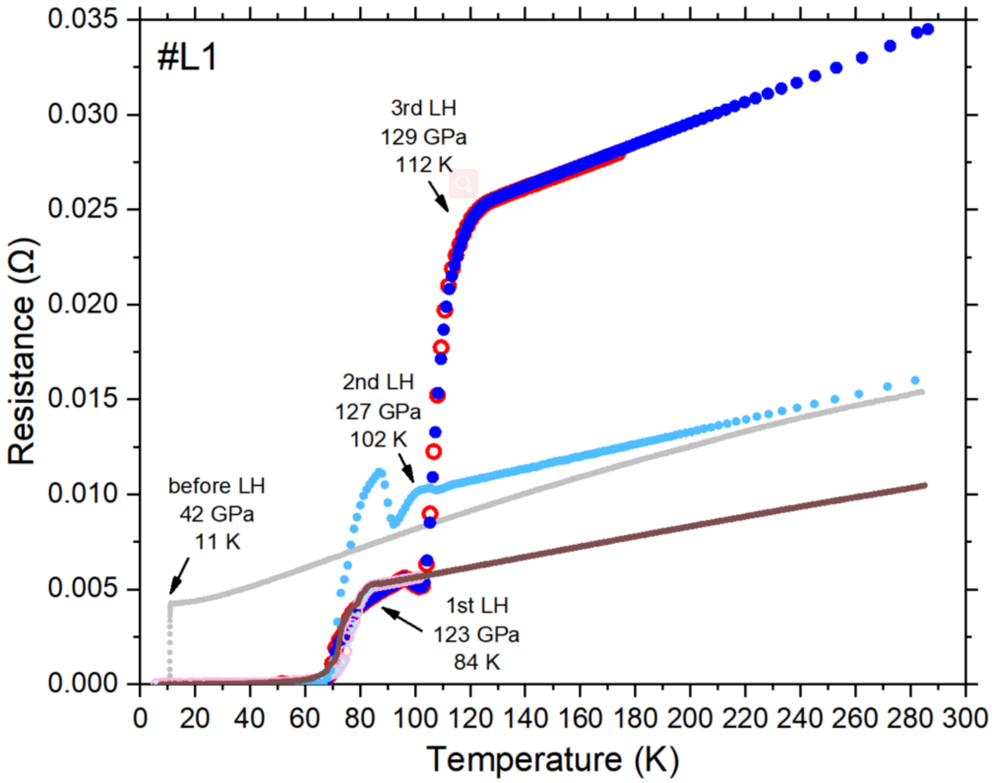

Fig. S30. Temperature dependence of the electrical resistance after 3 times laser-heating (LH). Solid circles represent the cooling cycle, and open circles represent the warming cycle.

Run #L2

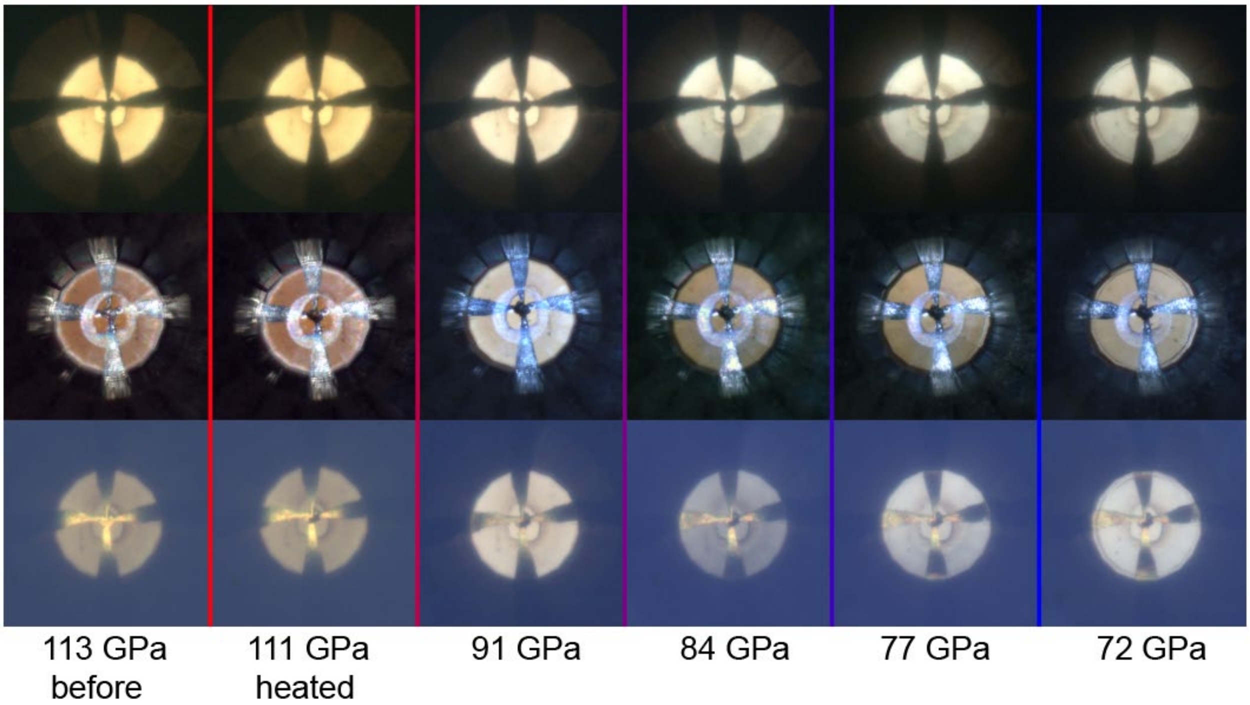

Fig. S31. Different photographic views of the diamond's bevel during the decompression of DAC #L2. In this run, we tested the nano-polycrystalline diamond (NPD)[3]. NPD consists of randomly oriented fine diamond nanocrystals and has higher Knoop hardness than SCD[4,5] .

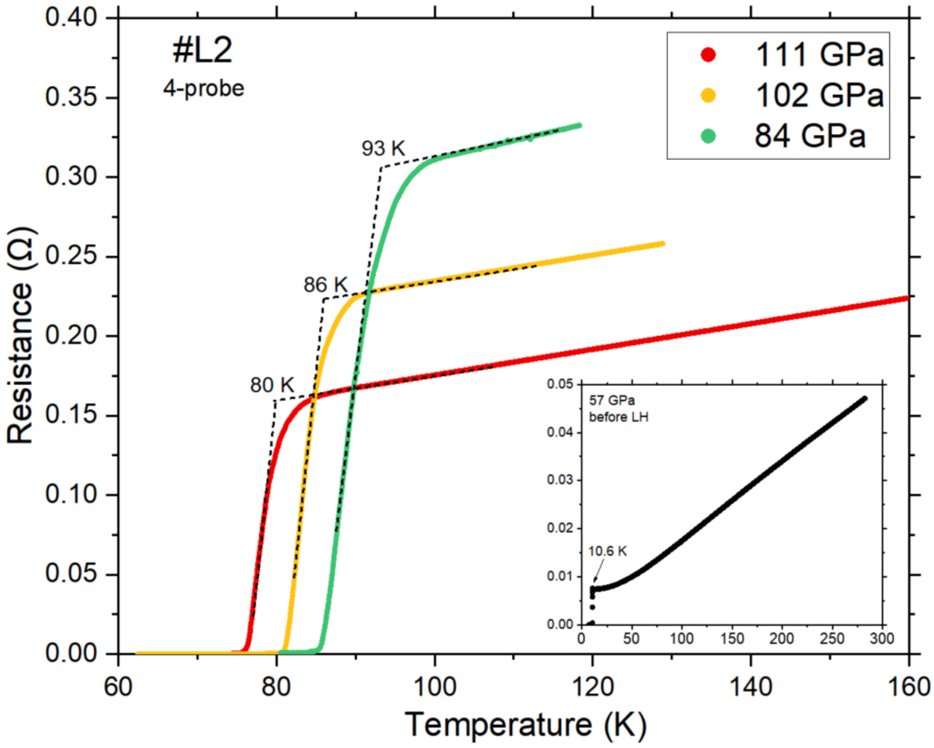

Fig. S32. Temperature dependence of the electrical resistance, measured using the four-probe method, showing the superconducting transitions of LaH_x in DAC #L2. Inset shows the superconducting transition of pure La at 57 GPa before the laser-heating.

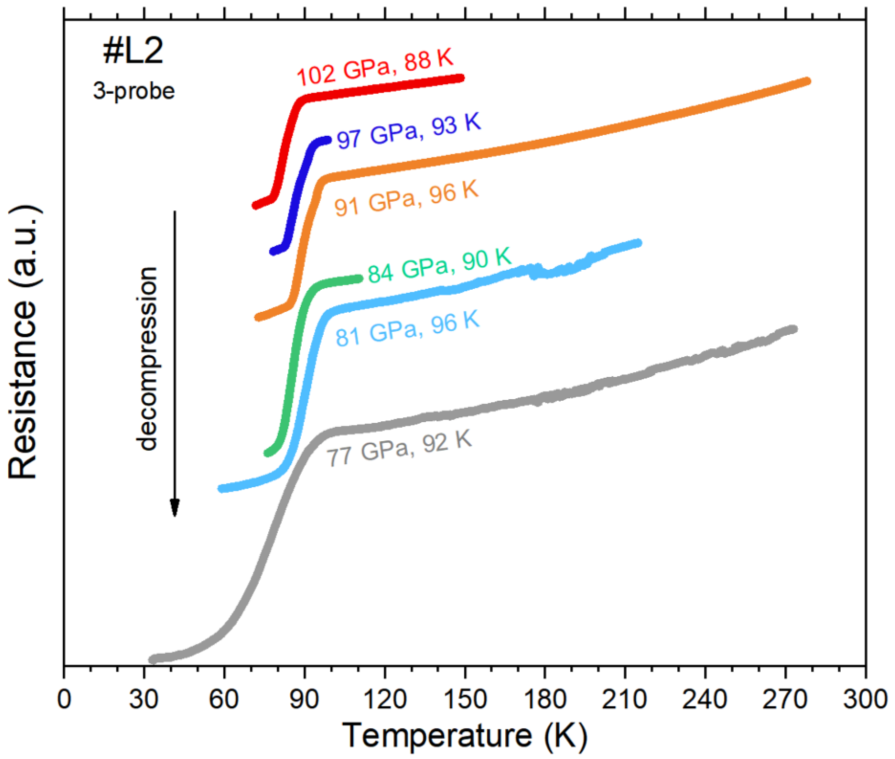

Fig. S33. Temperature dependence of the electrical resistance, measured using the three-probe method, showing the superconducting transitions of LaH_x in DAC #L2 during decompression.

Run #L3

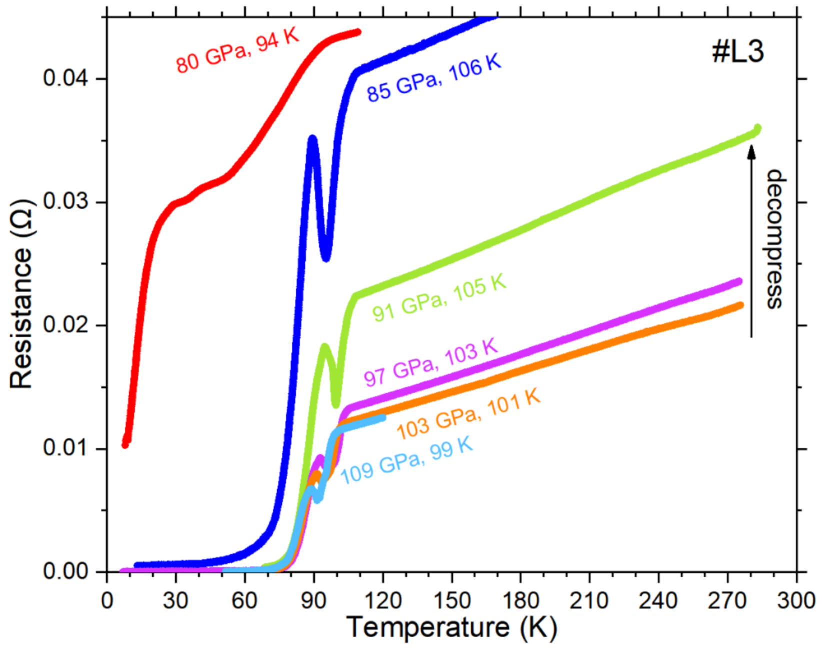

Fig. S34. Temperature dependence of the electrical resistance showing the superconducting transitions of LaH_x in DAC #L3.

Run #S

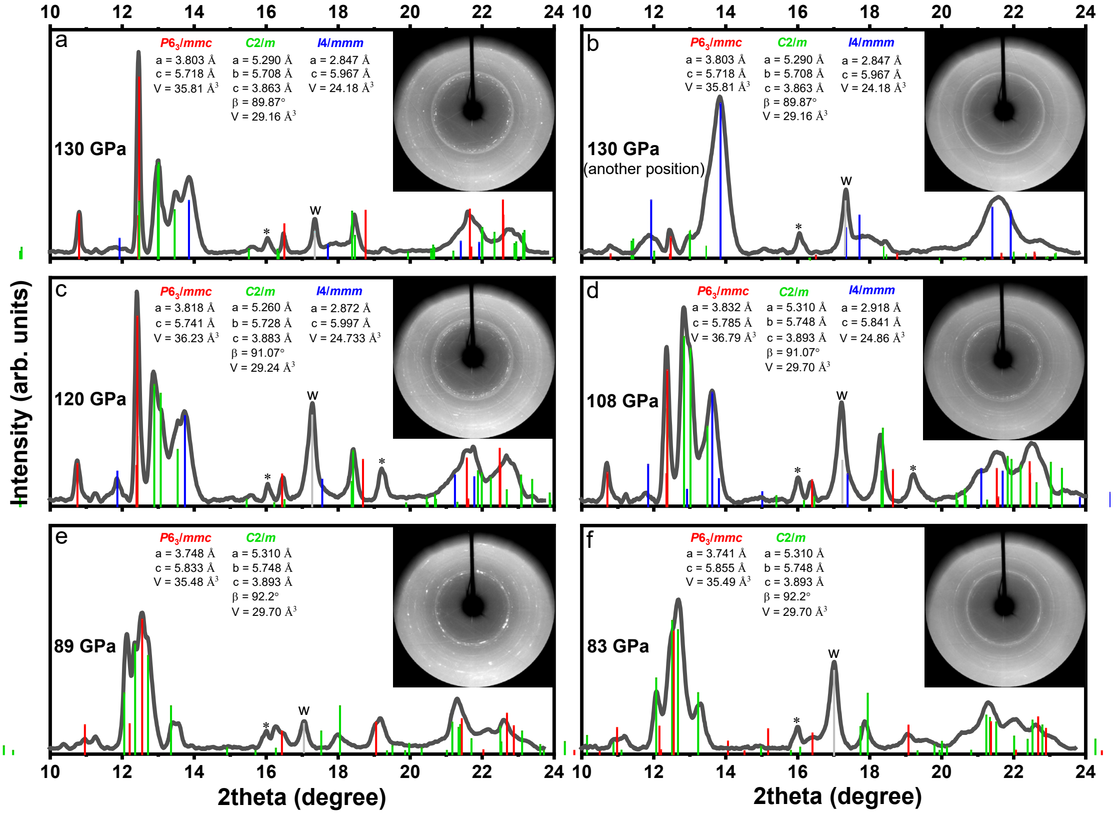

Fig. S35. Indexing of the synchrotron XRD data (λ = 0.6199 Å) for the sample in DAC #S after decompression to 130 GPa (a)-(b), 120 GPa (c), 108 GPa (d), 89 GPa (e) and 83 GPa (f), respectively. Insets show the XRD patterns.

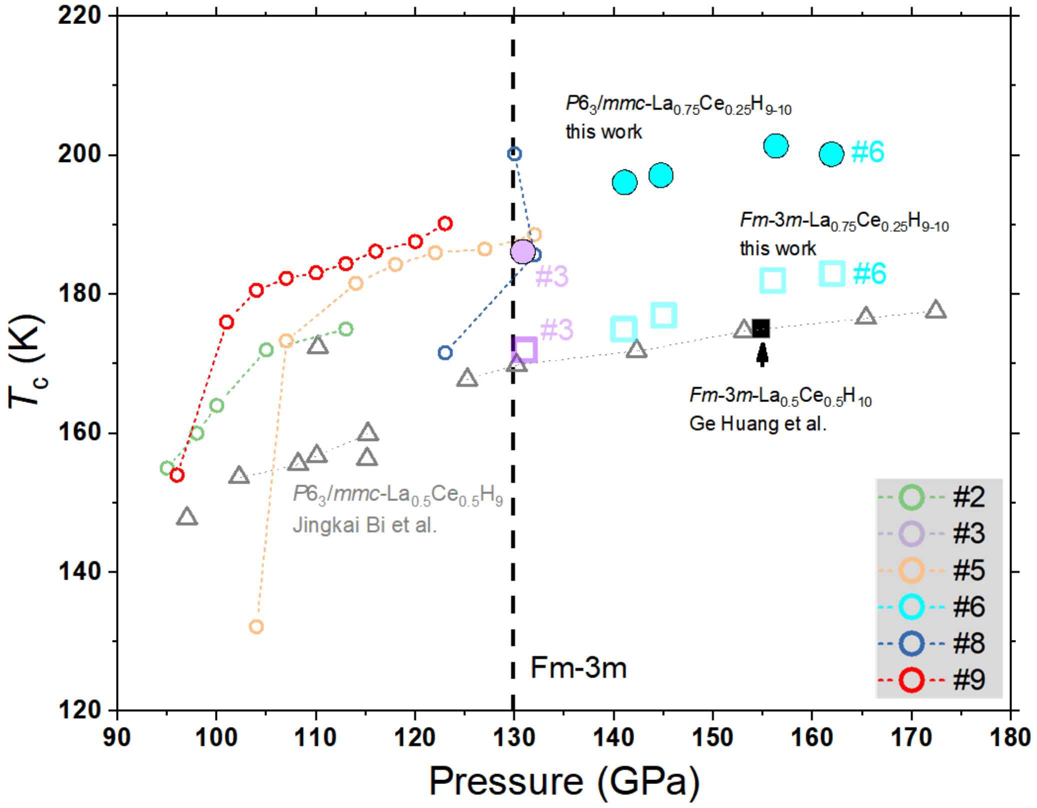

Fig. S36. Comparison of the T_c-P data with the results from another two similar works[6,7].

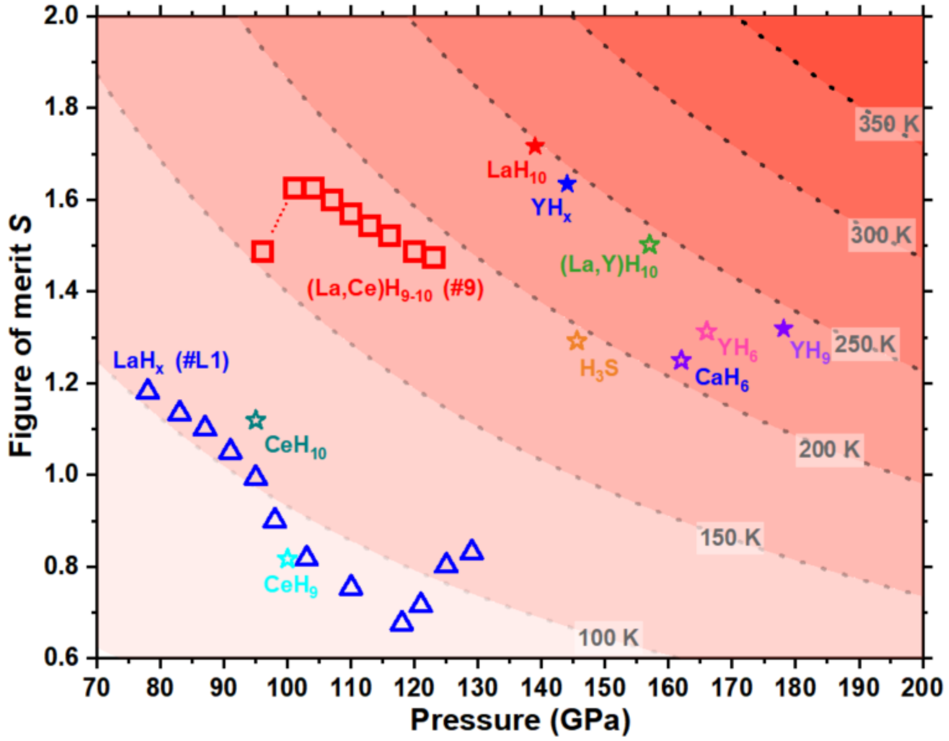

Fig. S37. *S*-*P* diagram of *hcp*-(La,Ce)H_{9-10} and LaH_x, including the experimentally highest *S* value of the well-known superconducting polyhydrides[8,9,10,11,12,13,14,15]. Solid symbols use the pressure scale of the hydrogen vibration. Dashed lines represent isotherms.

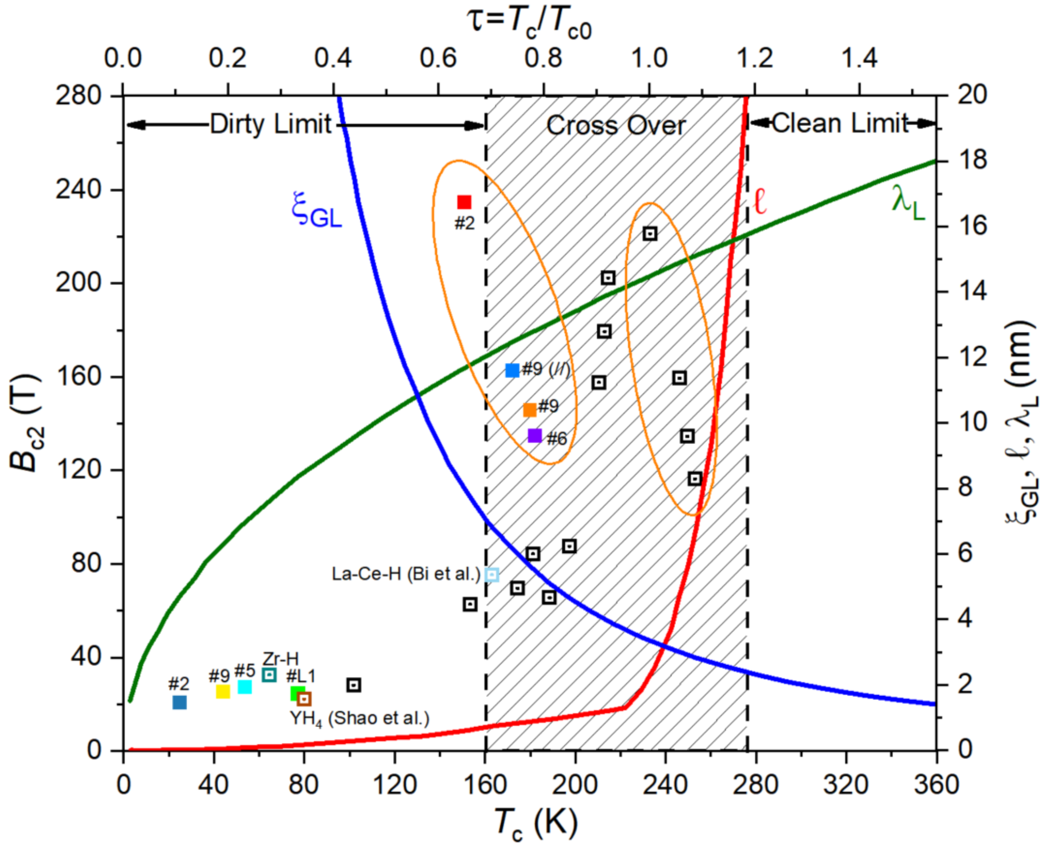

Fig. S38. The merged Fig. 1. (Various physical parameters from the GLAG theory applied to H-rich superconductors.) and Fig. 2. (The upper critical magnetic field B_{c2}) from Ref.[16], together with our data (colorful solid squares).

Analysis of the superconducting parameters

To get more information on the new superconducting La-Ce-H phase, we have estimated the superconducting parameters by carefully analyzing the temperature-dependent resistance $R(T)$ data. The Debye temperature θ_D of the synthesized La-Ce-H compound is calculated by fitting the experimental $R(T)$ data using the Bloch–Grüneisen (BG) formula[17], which has been used for some reported polyhydrides[18,19,20,21]:

$$R(T) = R_0 + A\left(\frac{T}{\theta_D}\right)^5 \int_0^{\frac{\theta_D}{T}} \frac{x^5}{(e^x - 1)(1 - e^{-x})} dx \qquad (1)$$

where A, θ_D, and R_0 were found using the least-squares method. These data also allow us to obtain the electron-phonon coupling parameter using the numerical reversal of the Allen-Dynes formula $T_c = F(\lambda, \omega_{log})$ →$\lambda = G(T_c, \omega_{log})$, where ω_{log} is replaced by $0.827 \times \theta_D$, and ω2 is 4/3 ω_{log} for simplicity. In other words, θ_D and ω_{log} are in the same ratio as the original McMillan formula and its subsequent modification by Allen and Dynes. We summarized the deduced parameters in Table S2 and plotted the data in Fig. S39. The fitted θ_D and ω_{log} remain around 700-900 K at 90-150 GPa, suggesting the need of relatively high λ = 2-3 to ensure that the observed T_c is above 160 K. Rather high λ corresponds to the weakly ordered, soft and highly defective structure of this superconducting La-Ce-H phase.

Table S2. Results of processing the dependence of electrical resistance on temperature according to the Bloch-Grüneisen formula for the cerium-lanthanum polyhydride (La,Ce)H_{9+x}. The resulting compound has a superconducting gap

DAC	Pressure, GPa	T_D, K	~ω_{log}, K ($0.827 \times T_D$)	λ	T_C, K (μ*=0.1)	Δ(0), meV
Cell 2	95	868	718	2.62	156	35.5
	98	750	620	3.26	161	38.1
	100	514	425	5.6*	164	37
Cell 3	131	750	620	3.29	165	39.2
	132	948	784	2.48	166	35.5
	140	878	726	2.72	166	38.2
Cell 6	150	1026	848	2.6	187	42.7
	156	1124	930	2.24	180	39.6
Cell 9	107	738	610	3.65	176	42.2
	93	587	485	1.41	59**	11.3
	123	1107	915	2.27	179	39.5
	113	662	547	4.42*	182	43.4
**theory**						
$LaCeH_{18}$	120	~704	582	3.16	159	38
La_3CeH_{36}	120	~827	684	2.16	131	28.8

* Probable phase transition

** Decomposition with losing hydrogen

Table S3. Results of processing the dependence of electrical resistance on temperature according to the Bloch-Grüneisen formula for the lanthanum polyhydrides LaH_x. Comparison of experimental and theoretical T_C allows us to estimate x < 9.

DAC	Pressure, GPa	T_D, K	~ω_{log}, K (0.827×T_D)	λ	T_C, K (μ*=0.1)
L1	129	546	451	3.11	115
	125	582	481	2.66	108
	121	504	417	2.54	90 [a]
	118	470	389	2.58	85 [a]
	110	516	427	2.41	88 [a]
	103	532	440	2.42	91
	98	574	475	2.36	96
	95	732	605	1.95	103
	91	778	643	1.87	105
	87	834	690	1.75	106
	78	1040	860	1.38 [b]	101
L2	111	446	369	2.60	81 [a]
	102	302	250	4.64 [c]	86
	84	472	390	2.86	93
L3	103	480	397	3.11	101 [a]
	97	524	433	2.82	102
	91	514	425	3.03	106
	85	562	465	2.65	104
**theory**					
*P*1-LaH_{10}	135	764	632	3.64	193
P6_3/*mmc*-LaH_9	120	447	370	4.73	150

[a] Linear temperature dependence R(T)

[b] Phase transition to stable phase with less hydrogen content

[c] Vicinity of phase transition

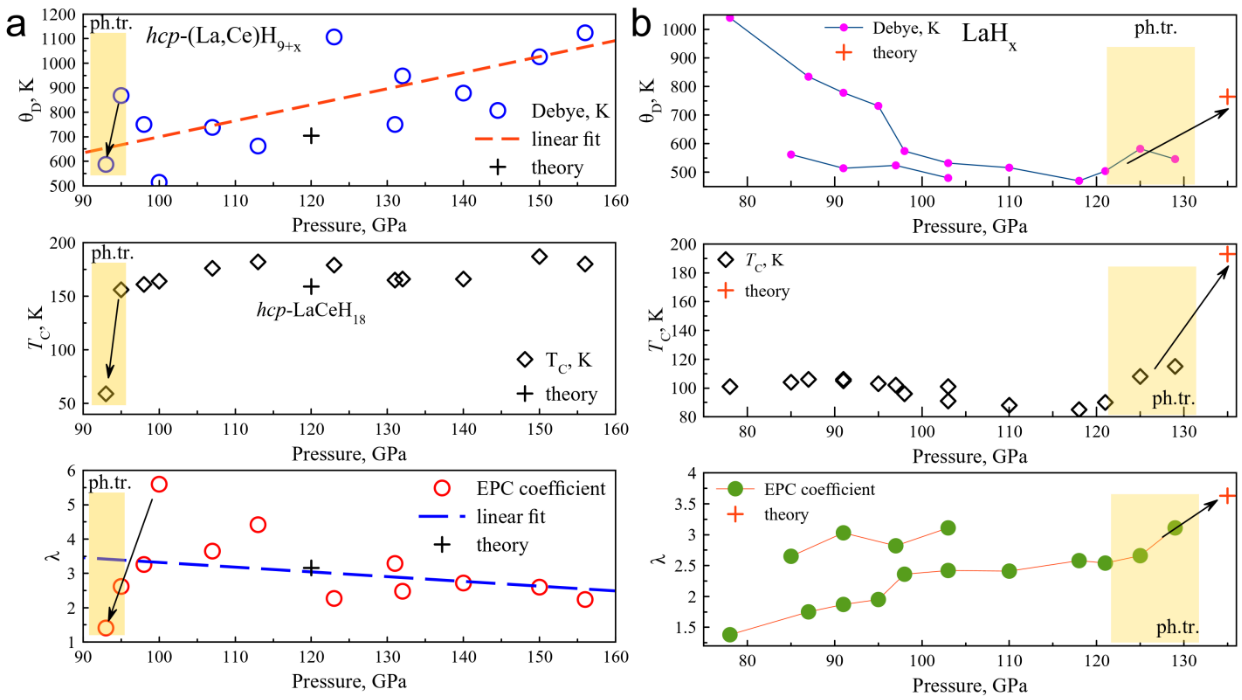

FIG. S39. Dependence of superconducting state parameters on pressure in La-Ce-H ternary hydrides (a) and La-H binary hydrides (b). Data is obtained using Bloch-Grüneisen and Allen-Dynes formulas. The onset of phase transformations ("ph.tr.") is highlighted by yellow strips. Simultaneous changes in all studied parameters are shown by arrows. "Theory" corresponds to QE calculations.

It is interesting that the dependence of the EPC parameter (λ) and the logarithmically averaged frequency (ω_{log}) are non-monotonic functions of pressure for the studied polyhydrides. It is generally assumed that the Debye temperature and ω_{log} will increase with increasing pressure. However, as Fig. S39b shows, this is not always the case. As the pressure increases, a phase transition to a more highly symmetric polyhydride modification may occur with a change in its stoichiometry. Such processes are usually accompanied by the appearance of "soft" phonon modes, a significant increase in the EPC coefficient, and a decrease in the average phonon energy.

In the cases studied, the phase transformations occur in the low-pressure interval of 90-100 GPa for Ce-La polyhydrides and in the high-pressure interval > 130 GPa for lanthanum hydrides. Simultaneous changes in all three parameters λ, T_C, ω_{log} and high values of λ correspond to these regions. In particular, for $(La,Ce)H_{9+x}$ (Fig. S39a), an abnormally high λ > 5 is observed at 100 GPa, which rapidly decreases to ≈1.5 at the pressure interval around 15 GPa, simultaneously with a sharp fall in the critical temperature of almost 3 times and a decrease in Debye temperature. This speaks in favor of a phase transformation with the loss of some hydrogen.

Theoretical Calculations

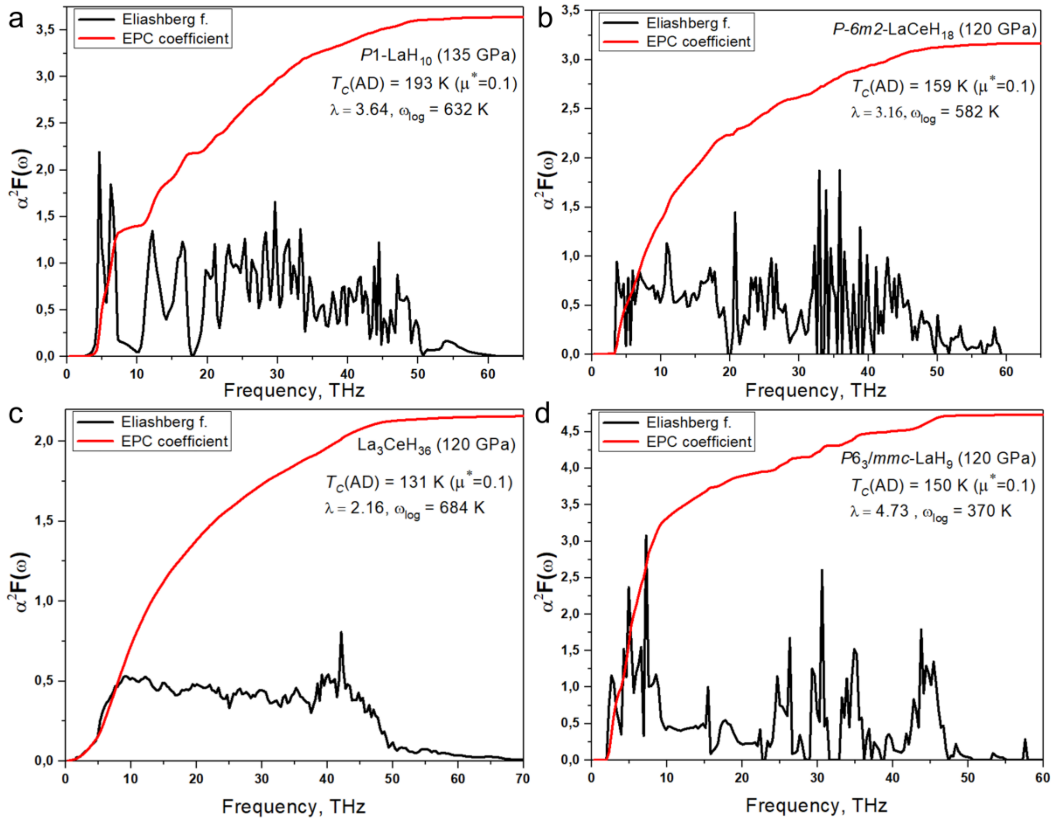

Fig. S40. Harmonic Eliashberg functions of studied La-Ce and La polyhydrides calculated within the tetrahedron method. (a) Distorted (*P*1) LaH_{10} at 135 GPa. (b) *P*-6*m*2-$LaCeH_{18}$ at 120 GPa. (c) Hexagonal La_3CeH_{36} at 120 GPa. (d) *P*6$_3$/*mmc*-LaH_9 at 120 GPa. Red curves correspond to the integrated EPC coefficient λ(ω). In the harmonic approximation, most of the compounds studied are unstable, so soft modes < 5 THz are excluded from the calculation.

The resulting Eliashberg functions require some discussion. As can be seen from Fig. S40d, the large values of the electron-phonon interaction coefficient (λ > 4.7) for hexagonal LaH_9 at 120 GPa indicate its dynamic instability[22]. This is one of the reasons why we do not observe the formation of this compound in the experiment. However, various dopants can stabilize a hexagonal *P*6$_3$/*mmc*-XH_9 structure at this pressure. One of them is Ce, which forms a stable hexagonal CeH_9[23,24,25]. Obviously, there is a certain critical concentration of cerium that stabilizes the hexagonal structure of XH_9. Both 25 at.% (this work) and 50 at.%[6] of cerium will definitely stabilize such a structure in the experiment. However, as we can see from Fig. S40c, the polyhydride with 25 at.% cerium has $\alpha^2F(\omega)$ characteristic of amorphous alloys and films (see for example[26,27]), which indicates the beginning of destruction of the ordered hexagonal structure. As the cerium concentration decreases to the critical value, the stability of hexagonal (La,Ce)H_9 will decrease, while the electron-phonon interaction coefficient (λ) and the critical temperature of superconductivity (T_C) will increase until (La,Ce)H_9

decomposes. This explains why the critical temperature for $La_{0.75}Ce_{0.25}H_9$ is greater than for $La_{0.5}Ce_{0.5}H_9$. An additional factor is also the suppression of superconductivity due to spin-flipping scattering on the local magnetic moments of Ce atoms[28].

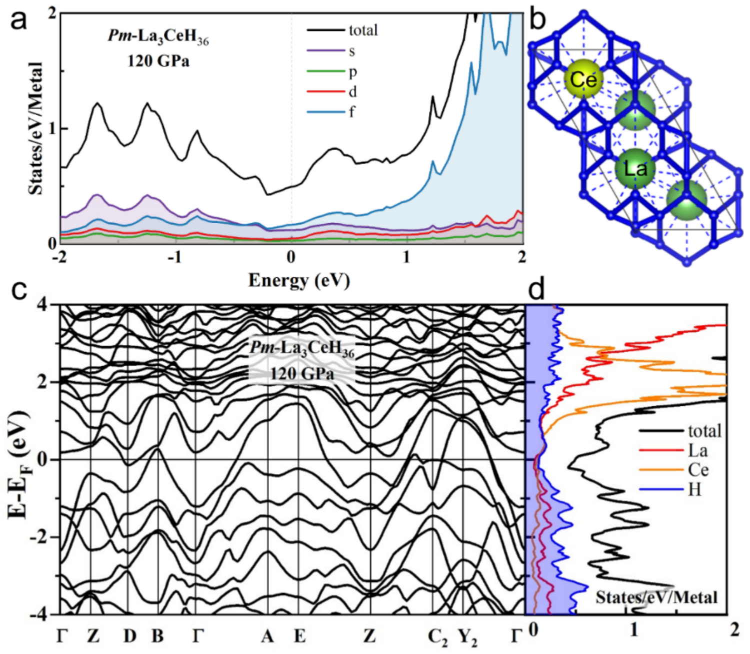

FIG. S41. Electronic properties of hexagonal La_3CeH_{36} at 120 GPa calculated using VASP (PAW PBE) code. (a) Partial contribution of different orbitals (s, p, d, f) to the density of states. (b) Structural model of model of the described compound. (c) Band structure of La_3CeH_{36}. vHs can be seen near "C_2". (d) Total electron density of states and the partial contribution of La, Ce and H atoms.

Note the relatively high contribution (0.17 states/eV/metal atom) of the *f*-electrons to the total density of states (DOS, 0.5 states/eV/metal atom), which results in a lower T_C than in pure $P6_3/mmc$-La_2H_{18} (Fig. S41). The contribution to the DOS from hydrogen is significant (0.12 states/eV/metal atom) and is at a similar level as from La and Ce. There is a van Hove singularity (vHs) in the vicinity of k(C_2) at the Fermi level. It is noteworthy that the introduction of only 25 at.% Ce provides the same density of states as 75 at.% La. However, there is no profit from that due to the increased Cooper pair-breaking on a small magnetic moment of Ce atoms. In addition, we should say the DOS changes in the vicinity of the Fermi level (decreasing by 15% in the interval of 200 meV), which limits the application of the constant DOS approximation[29]. Found values of the DOS and λ (experimental and theoretical) allow us to estimate the upper critical magnetic field $\mu_0H_{C2}(0)$[11,14] using an empirical formula (see Theoretical Methods), which has shown good results for many polyhydrides. We get $\mu_0H_{C2}(0) \sim$ 41-45 T (Table S4), much less than the experimental value (> 200 T), but quite close to the results of a similar investigation[6].

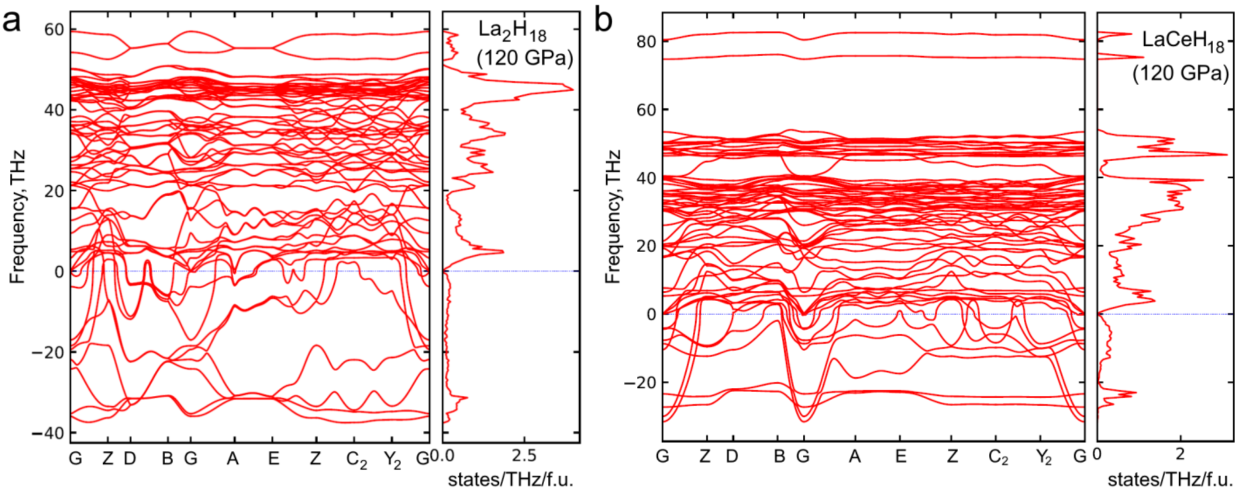

Fig. S42. Phonon band structure and density of states of distorted hexagonal (*P*6$_3$/*mmc*) La_2H_{18} (a) and $LaCeH_{18}$ (b) at 120 GPa calculated within harmonic approximation (f.u. = La_2H_{18} and $LaCeH_{18}$). Both compounds are dynamically unstable, but $LaCeH_{18}$ has shorter imaginary "tail" which allows us to say that cerium somewhat stabilizes the hexagonal XH_9 structure.

Many recent experimental studies of polyhydrides (e.g., LaH_{10}[30,31], $LaYH_{20}$[32], CeH_9[25], etc.) have shown that they are stable at much lower pressures than the dynamic stability criterion based on the harmonic approximation predicts. This serious discrepancy between the theoretical predictions and the experiment greatly devalues the harmonic calculations. We also investigated the applicability of Born's criteria[33,34] for hexagonal and cubic crystals under static isotropic pressure *P* to our polyhydrides. Table S5 shows that all compounds (LaH_{10}, $LaCeH_{18}$ and La_3CeH_{36}) are mechanically stable except *P*6$_3$/*mmc*-LaH_9, for which two criteria are not met.

Table S4. Parameters of the superconducting state of La-Ce and La polyhydrides calculated using the isotropic Migdal–Eliashberg equations (E)[35] and the Allen–Dynes formula (A–D)[36] with $\mu^* = 0.15–0.1$.

Pressure/compound	120 GPa *hcp*-$LaCeH_{18}$	120 GPa *hcp*-La_3CeH_{36}	120 GPa $P6_3/mmc$-La_2H_{18}	135 GPa $P1$-LaH_{10}
λ	3.16	2.16	4.73	3.64
ω_{log}, K	582	684	370	632
ω_2, K	987	1088	772	1037
α	0.48-0.49	0.47-0.49	0.48-0.49	0.48-0.49
T_C (A–D), K	146-159	117-131	141-150	179-193
T_C (E), K	172-187	136-150	164-178	215-231
$N(E_F)$, states/eV/metal*	0.82	0.77	0.79	0.78
$\Delta(0)$, meV	41.2-44.7	30.2-34.2	32.2-30	51-53.5
$\mu_0 H_C(0)$, T	57-59	41-45	45-44	67-68
$\Delta C/T_C$, mJ/mol·K^2	17.8-13.7	17.1-16.9	-	10.6-4.14
γ, mJ/mol·K^2	8.05	5.7	10.7	8.5
$R_\Delta = 2\Delta(0)/k_B T_C$	5.54	5.15-5.29	4.55-3.9	5.5-5.4

*Calculated using Quantum ESPRESSO code (pbe-spdn-kjpaw_psl pseudopotentials).

The calculation of the Debye temperature using elastic constants (Table S5) gives good agreement with the averaged experimental data for La_3CeH_{36} ($\theta_D(\exp) \approx 810$ K), but much worse for LaH_{10} which indicates that the experimentally synthesized compound in DACs L1-L3 is not $P1$-LaH_{10} and has a lower hydrogen content. Estimation of the average Fermi velocity $V_F \sim 4.76 \times 10^5$ m/s in hexagonal La_3CeH_{36} at 120 GPa makes it possible to estimate the London penetration depth of (La,Ce)H_9 polyhydrides $\lambda_L \sim 110$ nm, coherence length $\xi_{BCS} = 23$ nm, and lower critical magnetic field $\mu_0 H_{C1} = 0.09$ T. The calculated Ginzburg–Landau parameter[37] is over 46, which is typical for type-II superconductors.

Table S5. Elastic and thermodynamic parameters of lanthanum-cerium and lanthanum polyhydrides.

Pressure/compound	120 GPa *hcp*-$LaCeH_{18}$	120 GPa *hcp*-La_3CeH_{36}	120 GPa $P6_3/mmc$-La_2H_{18}	135 GPa $P1$-LaH_{10}
a, Å	3.726	3.763	3.758	3.590
c, Å	5.470	5.444	5.478	3.589
V_{DFT}, Å^3	65.60	135.68	67.15	34.46
C_{11}, GPa	517.9	629.9	461.7	532.1
C_{12}, GPa	279.1	165.5	388.5	175.9
C_{13}, GPa	204.7	180.3	186.1	198.3
C_{22}, GPa	552.7	470.0	483.6	557.3
C_{23}, GPa	195.6	167.8	162.6	234.7
C_{33}, GPa	497.9	482.2	480.2	534.03
C_{44}, GPa	66.8	130.6	13.3	211.1
C_{55}, GPa	9.1	19.3	99.5	241.2
C_{66}, GPa	119.4*	81.6	108.2	252.0
$C_{11}-\|C_{12}\| - P$, GPa	118.8	344.4	-46.9	221.2
$C_{33}(C_{11}+C_{12})-2C_{13}^2$, GPa^2	> 0	> 0	> 0	> 0
$C_{44} - P/2$, GPa	6.8	70.6	-46.7	143.6
B, GPa	322.6	282.0	305.6	314.8
G, GPa	61.0	88.4	62.1	201.1
E, GPa	172.1	238.4	172.9	497.3
Poisson ratio η	0.41	0.36	0.41	0.24
Debye temperature θ_D, K	637	761	642	1130
$\omega_{\log} = 0.827\theta_D$, K	527	629	531	935

* Calculated as $(C_{11}-C_{12})/2$ due to computational problems with convergence

Theoretical Methods

To calculate isotope coefficient α, the Allen-Dynes interpolation formulas were used:

$$\beta_{McM} = -\frac{d\ln T_C}{d\ln M} = \frac{1}{2}\left[1 - \frac{1.04(1+\lambda)(1+0.62\lambda)}{[\lambda - \mu^*(1+0.62\lambda)]^2}\mu^{*2}\right], \quad (2)$$

$$\beta_{AD} = \beta_{McM} - \frac{2.34\mu^{*2}\lambda^{3/2}}{(2.46+9.25\mu^*)\cdot((2.46+9.25\mu^*)^{3/2}+\lambda^{3/2})} - \frac{130.4\cdot\mu^{*2}\lambda^2(1+6.3\mu^*)\left(1-\frac{\omega_{\log}}{\omega_2}\right)\frac{\omega_{\log}}{\omega_2}}{\left(8.28+104\mu^*+329\mu^{*2}+2.5\cdot\lambda^2\frac{\omega_{\log}}{\omega_2}\right)\cdot\left(8.28+104\mu^*+329\mu^{*2}+2.5\cdot\lambda^2\left(\frac{\omega_{\log}}{\omega_2}\right)^2\right)}, \quad (3)$$

where the last two correction terms are usually small (~0.01).

The superconducting transition temperature T_C was estimated by Allen-Dynes formula in the following form:

$$T_C = \omega_{log}\frac{f_1 f_2}{1.2}\exp\left(\frac{-1.04(1+\lambda)}{\lambda-\mu^*-0.62\lambda\mu^*}\right), \tag{4}$$

where

$$f_1 f_2 = \sqrt[3]{1+\left(\frac{\lambda}{2.46(1+3.8\mu^*)}\right)^{\frac{3}{2}}}\cdot\left(1-\frac{\lambda^2(1-\omega_2/\omega_{log})}{\lambda^2+3.312(1+6.3\mu^*)^2}\right), \tag{5}$$

The EPC constant λ, logarithmic average frequency ω_{log} and mean square frequency ω_2 were calculated as:

$$\lambda = \int_0^{\omega_{max}} \frac{2\cdot\alpha^2 F(\omega)}{\omega} d\omega, \tag{6}$$

$$\omega_{log} = \exp\left(\frac{2}{\lambda}\int_0^{\omega_{max}}\frac{d\omega}{\omega}\alpha^2F(\omega)\ln(\omega)\right),$$

$$\omega_2 = \sqrt{\frac{1}{\lambda}\int_0^{\omega_{max}}\left[\frac{2\alpha^2F(\omega)}{\omega}\right]\omega^2 d\omega} \tag{7}$$

where μ^* is the Coulomb pseudopotential, for which we used widely accepted lower and upper bounds of 0.10 and 0.15.

The Sommerfeld parameter was found as

$$\gamma = \frac{2}{3}\pi^2 {k_B}^2 N(0)(1+\lambda), \tag{8}$$

where $N(0)$ – is the total density of electronic states at the Fermi level per spin. It was used to estimate the upper critical magnetic field and superconducting gap using the known semiempirical equations of the BCS theory (see Ref. [38], equations 4.1 and 5.11), working satisfactorily for $T_C/\omega_{log} < 0.25$:

$$\frac{\gamma' {T_C}^2}{(\mu_0 H_{C_2}(0))^2} = 0.168\left[1-12.2\left(\left(\frac{T_C}{\omega_{log}}\right)^2 ln\left(\frac{\omega_{log}}{3T_C}\right)\right)\right] \tag{9}$$

$$\frac{2\Delta(0)}{k_B T_C} = 3.53\left[1+12.5\left(\frac{T_C}{\omega_{\log}}\right)^2 \ln\left(\frac{\omega_{\log}}{2T_C}\right)\right] \tag{10}$$

where $\gamma' = 2\gamma$. Regarding formula (A8), the following remark should be made. The upper critical field by nature depends on the amount of impurities and defects in the sample, so it changes significantly from sample to sample. Only the lower limit for $\mu_0 H_{C2}$, which corresponds to an ideal crystal, can be estimated. However, we noticed that formula (S8), originally designed to calculate the thermodynamic critical field

μ_0H_C of superconductors, also gives a good estimate of the upper critical field of hydrides if the full DOS expressed per mole of a hydride is used in the Sommerfeld parameter (γ'). In this case, μ_0H_{C2} is expressed in Tesla in formula (S8).

The lower critical magnetic field was calculated according to the Ginzburg-Landau theory[39]

$$\frac{H_{C1}}{H_{C2}} = \frac{\ln\kappa}{2\sqrt{2}\kappa^2}, \quad \kappa = \frac{\lambda_L}{\xi} \tag{11}$$

where λ_L is the London penetration depth can be estimated by the formula

$$\lambda_L = 1.0541 \cdot 10^{-5}\sqrt{\frac{m_e c^2}{4\pi n_e e^2}} \tag{12}$$

here c - is the speed of light, e - is the electron charge, m_e - is the mass of an electron, and n_e - is an effective concentration of charge carriers expressed via the average Fermi velocity (V_F) in the Fermi-gas model:

$$n_e = \frac{1}{3\pi^2}\left(\frac{m_e V_F}{\hbar}\right)^3 \tag{13}$$

The average Fermi velocity can be estimated as

$$V_F = \frac{\pi \cdot \Delta(0)}{\hbar}\xi \tag{14}$$

where ξ is the coherence length calculated from the experimental upper critical magnetic field using the formula $\xi = \sqrt{\hbar / 2e(\mu_0 H_{C2})}$.

For processing the results of measurements of the critical temperatures in external magnetic fields we used the Werthamer-Helfand-Hohenberg (WHH) model simplified by Baumgartner et al.[40]

$$\mu_0 H_{C2}(T) = \frac{\mu_0 H_{C2}(0)}{0.693}\left(\left(1-\frac{T}{T_C}\right) - 0.153\cdot\left(1-\frac{T}{T_C}\right)^2 - 0.152\cdot\left(1-\frac{T}{T_C}\right)^4\right) \tag{15}$$

The critical temperature of superconducting transition was calculated using the Matsubara-type linearized Eliashberg equations[41]:

$$\hbar\omega_j = \pi(2j+1)k_B T, \qquad j = 0, \pm 1, \pm 2, \ldots \tag{16}$$

$$\lambda(\omega_i - \omega_j) = 2\int_0^\infty \frac{\omega \cdot \alpha^2 F(\omega)}{\omega^2 + (\omega_i - \omega_j)^2} d\omega \tag{17}$$

$$\Delta(\omega = \omega_i, T) = \Delta_i(T) =$$

$$= \pi k_B T \sum_j \frac{[\lambda(\omega_i - \omega_j) - \mu^*]}{\rho + |\hbar\omega_j + \pi k_B T \sum_k (sign\ \omega_k) \cdot \lambda(\omega_i - \omega_j)|} \cdot \Delta_j(T) \quad (18)$$

where T is the temperature in kelvins, μ^* is the Coulomb pseudopotential, ω is the frequency in Hz, $\rho(T)$ is a pair-breaking parameter, the function $\lambda(\omega_i - \omega_j)$ is related to an effective electron–electron interaction via the exchange of phonons [42]. The transition temperature can be found as the solution of the equation $\rho(T_C) = 0$, where $\rho(T)$ is defined as max(ρ), provided that $\Delta(\omega)$ is not a zero function of ω at a fixed temperature.

These equations can be rewritten in a matrix form as[43]

$$\rho(T)\psi_m = \sum_{n=0}^{N} K_{mn}\psi_n \Leftrightarrow \rho(T)\begin{pmatrix}\psi_1\\ \ldots\\ \psi_N\end{pmatrix} = \begin{pmatrix}K_{11} & \ldots & K_{1N}\\ \ldots & K_{\text{ii}} & \ldots\\ K_{N1} & \ldots & K_{\text{NN}}\end{pmatrix} \times \begin{pmatrix}\psi_1\\ \ldots\\ \psi_N\end{pmatrix}, \quad (19)$$

where ψ_n relates to $\Delta(\omega, T)$, and

$$K_{mn} = F(m-n) + F(m+n+1) - 2\mu^* - \delta_{mn}[2m + 1 + F(0) + 2\sum_{l=1}^{m} F(l)], \quad (20)$$

$$F(x) = F(x,T) = 2\int_0^{\omega\text{max}} \frac{\alpha^2 F(\omega)}{(\hbar\omega)^2 + (2\pi k_B T x)^2} \hbar\omega d\omega, \quad (21)$$

where $\delta_{nn} = 1$ and $\delta_{nm} = 0$ ($n \neq m$) is a unit matrix. Now we can replace the equation $\rho(T_C) = 0$ with the vanishing of the maximum eigenvalue of the matrix K_{nm}: [ρ = max_eigenvalue(K_{nm}) = $f(T)$, $f(T_C) = 0$].

In accordance with the Debye model[44,45], the Debye temperature (θ_D) and both sound velocities were calculated:

$$\theta_D = \frac{h}{k_B}\sqrt[3]{\frac{9n_{f.u.}V_{f.u.}}{4\pi(v_l^{-3} + 2v_t^{-3})}} = \frac{h\sqrt{N_A}}{k_B}\left\{\sqrt[3]{\frac{9n_{f.u.}}{4\pi}} \cdot \frac{V^{1/6}{}_{f.u.}}{\sqrt{M}} \cdot \frac{\sqrt{GB + (4/3)G^2}}{\sqrt[3]{2(B + (4/3)G)^{3/2} + G^{3/2}}}\right\}, \quad (S22)$$

$$v_l^{-3} = \left(\frac{\rho}{B + (4G/3)}\right)^{3/2}, \quad (S23)$$

$$v_t^{-3} = \left(\frac{\rho}{G}\right)^{3/2}, \quad (S24)$$

References

1. King, E., Harris, I. R. High pressure resistance measurements of some lanthanum-cerium alloys. *Journal of the Less Common Metals* **27**, 51-63 (1972).
2. Chen, W. et al. Superconductivity and equation of state of lanthanum at megabar pressures. *Phys. Rev. B* **102**, 134510 (2020).
3. Irifune, T., Kurio, A., Sakamoto, S., Inoue, T., Sumiya, H. Ultrahard polycrystalline diamond from graphite. *Nature* **421**, 599-600 (2003).
4. Bassett, W. A., Skalwold, E. A. Diamond cleavage: importance to high pressure research. *High Press. Res.* **37**, 46-58 (2017).
5. Odake, S., Ohfuji, H., Okuchi, T., Kagi, H., Sumiya, H., Irifune, T. Pulsed laser processing of nano-polycrystalline diamond: A comparative study with single crystal diamond. *Diamond Relat. Mater.* **18**, 877-880 (2009).
6. Bi, J. et al. Giant enhancement of superconducting critical temperature in substitutional alloy (La,Ce)H9. *Nat. Commun.* **13**, 5952 (2022).
7. Ge Huang, T. L., Philip Dalladay-Simpson, Liu-Cheng Chen, Zi-Yu Cao, Di Peng, Federico A. Gorelli, Guo-Hua Zhong, Hai-Qing Lin, Xiao-Jia Chen. Synthesis of Superconducting Phase of La0.5Ce0.5H10 at High Pressures. *Preprint at* https://doi.org/10.48550/arXiv.2208.05199 (2022).
8. Chen, W. et al. High-Temperature Superconducting Phases in Cerium Superhydride with a Tc up to 115 K below a Pressure of 1 Megabar. *Phys. Rev. Lett.* **127**, 117001 (2021).
9. Sun, D. et al. High-temperature superconductivity on the verge of a structural instability in lanthanum superhydride. *Nat. Commun.* **12**, 6863 (2021).
10. Einaga, M. et al. Crystal structure of the superconducting phase of sulfur hydride. *Nat. Phys.* **12**, 835-838 (2016).
11. Troyan, I. A. et al. Anomalous High-Temperature Superconductivity in YH6. *Adv. Mater.* **33**, 2006832 (2021).
12. Kong, P. et al. Superconductivity up to 243 K in the yttrium-hydrogen system under high pressure. *Nat. Commun.* **12**, 5075 (2021).
13. Snider, E. et al. Synthesis of Yttrium Superhydride Superconductor with a Transition Temperature up to 262 K by Catalytic Hydrogenation at High Pressures. *Phys. Rev. Lett.* **126**, 117003 (2021).
14. Semenok, D. V. et al. Superconductivity at 253 K in lanthanum–yttrium ternary hydrides. *Mater. Today* **48**, 18-28 (2021).
15. Ma, L. et al. High-Temperature Superconducting Phase in Clathrate Calcium Hydride CaH6 up to 215 K at a Pressure of 172 GPa. *Phys. Rev. Lett.* **128**, 167001 (2022).
16. Andrew, Keith, Salamat, A. Understanding Hydrogen Rich Superconductors: Importance of Effective Mass and Dirty Limit. *arXiv pre-print server*, (2022).
17. Adams, E. N. Electrons and phonons: J. M. Ziman: Clarendon Press, Oxford, 1960. 554 pp., 84s. *J. Phys. Chem. Solids* **15**, 359-360 (1960).
18. Talantsev, E. F. The electron–phonon coupling constant and the Debye temperature in polyhydrides of thorium, hexadeuteride of yttrium, and metallic hydrogen phase III. *J. Appl. Phys.* **130**, 195901 (2021).
19. Talantsev, E. F. Advanced McMillan's equation and its application for the analysis of highly-compressed superconductors. *Supercond. Sci. Technol.* **33**, 094009 (2020).
20. Talantsev, E. F., Mataira, R. C. Classifying superconductivity in ThH-ThD superhydrides/superdeuterides. *Mater. Res. Express* **7**, 016003 (2020).
21. Talantsev, E. F., Stolze, K. Resistive transition of hydrogen-rich superconductors. *Supercond. Sci. Technol.* **34**, 064001 (2021).
22. Yuzbashyan, E. A., Altshuler, B. L. Breakdown of the Migdal-Eliashberg theory and a theory of lattice-fermionic superfluidity. *Phys. Rev. B* **106**, 054518 (2022).
23. Salke, N. P. et al. Synthesis of clathrate cerium superhydride CeH9 at 80 GPa with anomalously short H-H distance. *Nat. Commun.* **10**, 4453 (2019).
24. Li, X. et al. Polyhydride CeH9 with an atomic-like hydrogen clathrate structure. *Nat. Commun.* **10**, 3461 (2019).
25. Chen, W. et al. High-Temperature Superconducting Phases in Cerium Superhydride with a Tc up to 115 K below a Pressure of 1 Megabar. *Phys. Rev. Lett.* **127**, 117001 (2021).

26. Bergmann, G. Amorphous metals and their superconductivity. *Phys. Rep.* **27**, 159-185 (1976).
27. Garland, J. W., Bennemann, K. H., Mueller, F. M. Effect of Lattice Disorder on the Superconducting Transition Temperature. *Phys. Rev. Lett.* **21**, 1315-1319 (1968).
28. Semenok, D. V. et al. Effect of Magnetic Impurities on Superconductivity in LaH10. *Adv. Mater.*, 2204038 (2022).
29. Sano, W., Koretsune, T., Tadano, T., Akashi, R., Arita, R. Effect of Van Hove singularities on high Tc superconductivity in H3S. *Phys. Rev. B* **93**, 094525 (2016).
30. Drozdov, A. P. et al. Superconductivity at 250 K in lanthanum hydride under high pressures. *Nature* **569**, 528-531 (2019).
31. Somayazulu, M. et al. Evidence for Superconductivity above 260 K in Lanthanum Superhydride at Megabar Pressures. *Phys. Rev. Lett.* **122**, 027001 (2019).
32. Semenok, D. V. et al. Superconductivity at 253 K in lanthanum-yttrium ternary hydrides. *Materials Today* **48**, 18-28 (2021).
33. Mouhat, F., Coudert, F.-X. Necessary and sufficient elastic stability conditions in various crystal systems. *Physical Review B* **90**, 224104 (2014).
34. Karki, B. B., Ackland, G. J., Crain, J. Elastic instabilities in crystals from ab initio stress - strain relations. *Journal of Physics: Condensed Matter* **9**, 8579 (1997).
35. Eliashberg, G. M. Interactions between Electrons and Lattice Vibrations in a Superconductor. *JETP* **11**, 696–702 (1959).
36. Allen, P. B., Dynes, R. C. Transition temperature of strong-coupled superconductors reanalyzed. *Phys. Rev. B* **12**, 905-922 (1975).
37. Ginzburg, V. L., Landau, L. D. On the Theory of superconductivity. *Zh. Eksp. Teor. Fiz.* **20**, 1064-1082 (1950).
38. Carbotte, J. P. Properties of boson-exchange superconductors. *Rev. Mod. Phys.* **62**, 1027-1157 (1990).
39. Ginzburg, V. L., Landau, L. D., Eksp., Z., Fiz., T. Collected Papers of L.D. Landau (Oxford: Pergamon Press, 1965) p. 546. (1965).
40. Baumgartner, T., Eisterer, M., Weber, H. W., Flükiger, R., Scheuerlein, C., Bottura, L. Effects of neutron irradiation on pinning force scaling in state-of-the-art Nb3Sn wires. *Superconductor Science and Technology* **27**, 015005 (2013).
41. Eliashberg, G. M. Interactions between electrons and lattice vibrations in a superconductor. *Sov. Phys. JETP* **11**, 696-702 (1959).
42. Bergmann, G., Rainer, D. The sensitivity of the transition temperature to changes in α2F(ω). *Zeitschrift für Physik* **263**, 59-68 (1973).
43. Allen, P., Dynes, R. A computer program for numerical solution of the Eliashberg equation to find Tc. *Technical Report* **7** TCM/4/1974 (1974).
44. Debye, Peter. Zur Theorie der spezifischen Waerme. *Annalen der Physik* **39**, 789-839 (1912).
45. Anderson, O. L. Determination and Some Uses of Isotropic Elastic Constants of Polycrystalline Aggregates Using Single-Crystal Data. *In Physical Acoustics* **3B**, 43-95 (1965).
